# *Acinetobacter guillouiae*, a lipolytic strain isolated from sludge capable of partially depolymerising polyethylene terephthalate: genomic, proteomic, and biochemical insights

**DOI:** 10.1186/s12866-026-05567-7

**Published:** 2026-08-26

**Authors:** Naheed Akhtar, Afef Najjari, Anna Magnone, Esma Ceylan Yerebakan, Katja Bernfur, Cecilia Tullberg, Muhammad Siddique Awan, Zahid Majeed, Carl Grey, Baozhong Zhang, Javier A. Linares-Pastén

**Affiliations:** 1 Division of Biotechnology and Applied Microbiology, Department of Process and Life Sciences Engineering, Faculty of Engineering (LTH), Lund University, P.O. Box 124, Lund, SE- 22100 Sweden; 2 Department of Zoology, The University of Azad Jammu and Kashmir, Muzaffarabad, Pakistan; 3 Laboratoire de Microbiologie et Biomolécules Actives, Université Tunis El Manar, Faculté des Sciences de Tunis, Tunis, 2092 Tunisie; 4 Department of Biology, Faculty of Sciences, Karadeniz Technical University, Trabzon, 61080 Turkey; 5 Division for Biochemistry and Structural Biology, Department of Chemistry, Lund University, P.O. Box 124, Lund, SE-22100 Sweden; 6 Department of Biotechnology, The University of Azad Jammu and Kashmir, Muzaffarabad, Pakistan; 7 Centre for Analysis and Synthesis, Department of Chemistry, Faculty of Engineering (LTH), Lund University, P.O. Box 124, Lund, SE-22100 Sweden

**Keywords:** Partial PET-depolymerisation, *Acinetobacter*, Sludge, Lipases, Esterases

## Abstract

*Acinetobacter guillouiae* I-MWF was isolated by incubating amorphous polyethylene terephthalate (PET) film in sludge samples. The strain partially depolymerised PET powder with 11.3% crystallinity, as confirmed by FT-IR, HPLC-UV, and LC-MS analyses. Extracellular enzymes released terephthalic acid (TPA), mono(2-hydroxyethyl) terephthalate (MHET), and bis(2-hydroxyethyl) terephthalate (BHET). Genomic analysis identified 18 putative extracellular hydrolases, including lipases and esterases, each with a conserved catalytic triad. Proteomic profiling revealed expression of two triacylglycerol lipases and two additional lipase-family proteins when the strain was cultivated with PET or a PET–Tween 80 mixture. These enzymes were cloned in *Escherichia coli*, but most formed insoluble, inactive inclusion bodies, and one was not expressed. Molecular modelling highlighted structural features likely to influence their catalytic interaction with PET. Although the strain partially depolymerised PET powder, it was unable to grow on PET, TPA, or ethylene glycol, indicating that PET depolymerisation occurs as a side activity rather than supporting growth. Instead, *A. guillouiae* displayed strong lipolytic activity and a clear preference for lipid-based substrates, achieving its highest growth with Tween 80. A lipid transporter was also expressed under these conditions, suggesting adaptation to hydrocarbon-rich environments. These findings indicate that *A. guillouiae* I-MWF can mediate partial PET depolymerisation without assimilating the resulting monomers, while preferentially growing on lipid-like substrates.

**Supplementary Information:**

The online version contains supplementary material available at 10.1186/s12866-026-05567-7.

## Introduction

Global plastic pollution, particularly from single-use plastic products, has significantly increased research on plastic recycling and degradation in recent years. In this context, biological technologies have emerged as one of the most attractive alternatives to chemical and mechanical processes because of their mild reaction conditions, environmentally friendly procedures, and the potential to become a sustainable solution [1]. Some organisms can metabolise plastic depolymerisation products, such as monomers, which can be converted into commercial products by fermentation [2].

Most plastic-active enzymes have been found in bacteria and fungi. Recently, the first archaeal polyethene terephthalate (PET) depolymerising enzyme was reported [3]. Bioinformatic analyses of global microbiomes have identified 30,000 genes encoding putative enzymes capable of degrading 10 different types of plastic [4]. PET is one of the most widely used plastics and accounts for 10 to 20% of global plastic pollution. A bioinformatic study reported 504 PET-active enzymes in bacteria, mainly in the phyla *Actinobacteria*, *Proteobacteria*, and *Bacteroidetes* [5]. However, fewer than 50 enzymes with hydrolytic activity against PET and polyurethane have been experimentally verified, most of which are PET-active [6].

Plastics are typically recalcitrant materials. The chemical structure, considerable molecular weight, and crystallinity are the main obstacles to their biological degradation. Despite these properties, plastics with heteroatom backbones, such as polyesters and polyurethanes, are more susceptible to enzymatic attack than those with polyolefin backbones, such as polyethylene, polypropylene, and their derivatives [7]. For example, polyesters and polyurethanes have ester and amide bonds, respectively, which are also widely present in biological molecules. Therefore, some hydrolases, such as esterases and amidases, could hydrolyse these types of bonds. PET is the most studied polyester in terms of enzymatic depolymerisation [8]. However, in most cases, PET pretreatment is necessary to amorphize high-crystalline domains, thereby making the plastic more accessible to enzymatic treatment. One strategy is to use thermostable PET-depolymerising enzymes that can operate near PET’s glass transition temperature (67–81˚C), which are among the most effective [9].

So far, the enzymes capable of hydrolysing PET are serine hydrolases, including cutinases, carboxylesterases, and lipases [10]. The most studied PET hydrolases are *Is*PETase from *Ideonella sakaiensis* [11] and the leaf-branch compost cutinase (LCC) [12]. These enzymes and related cutinases depolymerise PET into terephthalic acid (TPA), mono(2-hydroxyethyl) terephthalic acid (MHET), bis(2-hydroxyethyl) terephthalic acid (BHET), and ethylene glycol. The product profile can vary with enzyme concentration, reaction time, or the specific cutinase; however, MHET is usually the dominant product, followed by TPA and a minor amount of BHET.

Beyond their biotechnological potential, plastic-degrading microorganisms may also play a role in nature. Their ability to interact with plastics indicates that some microbes are beginning to adapt to human-made pollution, especially in environments constantly exposed to plastic waste. In this context, the study investigates bacteria in activated aerobic sludge that can interact with PET. For this purpose, a collection of strains was isolated using a two-step approach: selecting plastic-colonising microorganisms and assessing their capacity to depolymerise PET powder in a mineral salt culture medium. One of the isolated organisms, identified as *Acinetobacter guillouiae*, was the most promising strain, capable of partially depolymerising PET powder. This strain, named I-MWF, was isolated from sludge samples collected at a water filtration plant in Makri, Muzaffarabad, on the Neelum River in Azad Jammu and Kashmir, Pakistan. Household and commercial waste from these urban and rural populations of 0.7 million produces tons of plastic waste that enter the riverine water.

Several *Acinetobacter* species have attracted attention for their role in bioremediation. In the PlasticDB database [13], a related species, *A. baumannii*, with strains isolated from landfills, soil, and plastic waste dumping sites, showed potential for PET depolymerisation. However, reported evidence of biodegradation relies on indirect methods such as FT-IR, weight loss, SEM, or CO_2_ production. In this work, we report enzymatic activity against PET from extracellular extracts obtained during the cultivation of *A. guillouiae* I-MWF in the presence of PET powder. HPLC-UV/MS confirmed enzymatic depolymerisation of PET and its products. Furthermore, genomic and proteomic analyses have revealed the expression of lipases, suggesting their involvement in the partial hydrolysis of polyesters. The molecular structures of these enzymes were also investigated in silico, providing insights into their potential interactions with PET substrates.

## Materials and methods

### Chemicals

All chemicals were obtained from Sigma-Aldrich (USA). Amorphous polyethylene terephthalate (PET) film was sourced from Goodfellow (Germany). PET Ramapet N180 was purchased from Indorama and prepared as a semicrystalline powder via solubilization and precipitation, as previously described [14]. The crystallinity, previously determined by differential scanning calorimetry, was 11.3% [15].

### Cultivation media

All chemicals used were of analytical grade and purchased from Sigma-Aldrich. Minimal Salt Medium (MSM) and Lysogeny Broth (LB) were used in this study. MSM contained 0.2 g KH_2_PO_4_, 1 g K_2_HPO_4_, 1 g NaCl, 0.002 g CaCl_2_.H_2_O, 0.5 g MgSO_4_.7H_2_O, 1 g (NH_4_)_2_SO_4_, and 0.01 g FeSO_4_.7H_2_O per litre. LB was prepared with 10 g tryptone, 5 g yeast extract, and 5 g NaCl in 1 L. MSM and LB agar plates were prepared by adding 16 g of agar to 1 L of distilled water.

### Sampling

The sample consisted of aerobic activated sludge collected from the filtration bed of a water treatment plant in Makri, Muzaffarabad (AJK), Pakistan. The geographic coordinates are 34.39˚ latitude and 73.47˚ longitude. Sludge samples were collected from a publicly accessible water treatment facility, and no specific permission was required.

### Bacterial strain isolation

The source sample was aliquoted in volumes of 10 mL, and a small piece (approx. 1.5 cm × 1 cm, and 0.02 mm thickness) of amorphous PET (Goodfellow) was submerged in each aliquot. Before use, the films were washed and sterilised with 70% ethanol. The samples were incubated statically at room temperature for 7 days. Then, the pieces of plastic were aseptically removed, washed with 0.9% NaCl, transferred to an MSM medium, and incubated for 7 days at room temperature to capture potential plastic degraders that colonised the plastic films. Growth was monitored by measuring optical density (OD) at a wavelength of 600 nm. Then, the colonies were isolated by serial dilutions and spread on LB agar plates. The cultivation in a solid medium was incubated at 23˚C for 48 h. Isolated bacterial strains were transferred to LB broth with glycerol (20%) and stored at − 80˚C for further analysis.

### Optimisation of different parameters for the growth of isolated bacteria

Inoculum size, pH, and temperature were optimised. All cultivations were performed in triplicate using 100 mL of MSM containing 20 mg of PET powder. The temperature range was 23 to 43 °C, pH 6 to 8, and inoculum volumes of 1, 1.5, and 2 mL. Cultivations were incubated with shaking at 120 rpm. ODs at 600 nm were measured every 24 h for 3 days. The PET powder was sterilised by rinsing with 70% ethanol before use. The pH was adjusted to 7.5 using 1 M NaOH or HCl before autoclaving.

### Bacterial activity against PET

The ability of the isolated bacteria to degrade PET was assessed by monitoring their growth in MSM medium supplemented with PET powder as the sole carbon source for 28 days. All experiments were carried out in triplicate, with controls consisting of MSM without bacterial inoculum. Cultures were prepared in 100 mL of MSM containing 20 mg of PET powder and incubated at 23 °C with shaking at 120 rpm. Growth was monitored spectrophotometrically by measuring the OD at 600 nm.

### Growth curves

Growth curves were generated using different carbon substrates. All cultivations were performed in 100 mL of MSM containing 20 mg of PET powder, 5 mg TPA, 0.1-0.5 mL ethylene glycol, 20 mg of chitin, 20 mg of cellulose, 1 mL of Tween 80, 1 mL of olive oil, or 200 mg of glucose as the sole carbon source. Cultures were carried out in triplicate and incubated in shake flasks at 23 °C for 24 h. Growth was monitored by measuring OD at 600 nm. Specific growth rates (µ_max_) and generation times (t_g_) were determined for each cultivation.

### Extracellular enzymes recovery

The cells were cultured in 200 mL of MSM with 20 mg of PET powder as a substrate at 23˚C for 15 days. Subsequently, the cell pellet and residual PET were separated by centrifugation at 4500 × g for 20 min. The extracellular fraction was concentrated using spin concentration tubes with 10 kDa cut-off membranes. Total protein concentration was determined using the bicinchoninic acid (BCA) assay kit, according to the manufacturer’s protocol (Sigma-Aldrich).

### Enzyme activity assay

Enzymatic activity on PET powder was assessed using cell-free extracts obtained from culture supernatants. Concentrated extracellular fractions were subjected to an enzyme activity assay on PET powder. Twenty milligrams of semicrystalline polymer powder was soaked in 1 mL of the extracellular fraction, and the reaction mixture was incubated at 23 °C and 200 rpm for 72 h. The depolymerisation products were then analysed by HPLC-UV/MS.

PET-hydrolytic activity was estimated using purified *Is*PETase from *Ideonella sakaiensis*, produced and purified in-house, as a reference enzyme [14]. *Is*PETase concentrations ranging from 3.75 to 63.5 µg/mL were incubated with PET under the same assay conditions used for extracellular samples.

Because standards for MHET and BHET were unavailable, all PET depolymerisation products were quantified using a TPA calibration curve. Consequently, the concentrations of TPA, MHET, and BHET are TPA-equivalent, based on their chromatographic responses. The total concentration of soluble aromatic PET hydrolysis products was calculated as the sum of the individual TPA-equivalent concentrations and is reported as TPA equivalents (${TPA}_{eq}$).

The relationship between soluble aromatic product formation and enzyme loading was described using a Hill-type saturation model:

$$\:P=\:{P}_{max}\:\frac{{x}^{n}}{{K}^{n}+\:{x}^{n}}$$

where * x* is the enzyme-to-PET ratio (E/PET, mg enzyme/mg PET), * P* is the concentration of soluble aromatic products expressed as ${TPA}_{eq}$, $\:{P}_{max}$ is the maximum $\:{TPA}_{eq}$ concentration attainable under the assay conditions, * K* is the E/PET ratio corresponding to half-maximal product formation, and * n* is the Hill coefficient.

Apparent PETase-equivalent activity and PET depolymerisation percentages of extracellular samples from *A. guillouiae* were estimated by interpolating the measured $\:{TPA}_{eq}$ values on the fitted *Is*PETase calibration curve.

### HPLC and LC-MS Analysis

The products resulting from enzymatic degradation were analysed using the HPLC-UV/MS system [16]. The HPLC setup employed was the Dionex Ultimate 3000 RS, which is equipped with a UV/Vis detector (SPD-20 A). The LC-MS system used was the ThermoFisher Scientific Ultimate 3000 RS HPLC system, integrated with an LTQ Velos Pro Ion trap mass spectrometer and a heated electro-spray ionisation source (HESI-II). The UV analysis was performed at 260 nm, while the mass spectrometer operated in negative ion mode, covering a mass range from m/z 50 to 700. The HESI source was set at 300˚C with a spray voltage of 3.0 kV. Helium served as the collision gas, and nitrogen as the nebulising gas. For LC-MS/MS, data-dependent acquisition (DDA) was used with a CID fragmentation energy setting of 35%. Chromatographic separation was achieved using a hydrophobic C_18_ column (Kinetex^®^ 1.7 μm XB-C_18_, 100 Å, LC Column 50 × 2.1 mm) with a mobile phase consisting of a 20% acetonitrile and 80% formic acid (0.1%) mixture at a constant flow rate of 0.4 mL/min for 3 min.

### 16 S rRNA gene sequencing and phylogenetic assignment

Genomic DNA was extracted using the GeneJET™ Genomic DNA Purification Kit (Thermo Scientific). The 16S gene was PCR amplified using the universal primers 16S-FD1 (5’ GAC TTT GAT CCT GGC TCA-3’) and 16S-RP2 (5’ ACG GCT AAC TTG TTA CGA CT-3’). PCR reactions were performed with iPoofTM HF kit (Bio-Rad, USA) using 50 ng of genomic DNA as a template in 20 µL of the reaction mix. The amplification consisted of 34 cycles after an initial DNA denaturation at 95˚C for 3 min and before a final polymerization at 72˚C for 5 min. Each cycle included denaturation at 95˚C for 30 s, annealing at 55˚C for 30 s, and extension at 72˚C for 1 min. The products were purified using the GeneJET PCR Purification Kit (#K0702; Thermo Scientific, USA), and their quality and concentration were evaluated by agarose gel electrophoresis and UV spectroscopy, respectively. The amplicons were sequenced at Eurofins, Germany.

The isolated bacterial strain I-MWF was identified based on its 16 S rRNA gene sequence. Sequence similarity searches were performed using BLAST against the EzBioCloud (www.ezbiocloud.net - formerly Ez-Taxon) database and the DDBJ/NCBI databases. Closely related sequences corresponding to type strains were retrieved from EzBioCloud for phylogenetic analysis. A phylogenetic tree of *Acinetobacter* strains was constructed using the neighbour-joining method with the Tamura–Nei nucleotide substitution model [17]. Evolutionary analyses were conducted in MEGA X v10.2.6 using ClustalW for sequence alignment [18], and the robustness of the tree was evaluated by bootstrap analysis with 1000 replicates [19].

### Genome sequencing, assembly, annotation, phylogenomics and comparative genomics analysis

Whole-genome DNA sequencing of *A. guillouiae* I-MWF was performed using GS on an Illumina NovaSeq PE150 platform (Illumina Inc.) at Novogene (China). Sequence read quality control was first assessed using FastQC version 0.10.0 [20]. The reads were then trimmed using Trimmomatic [21] and assembled with SPAdes v.3.13 [22]. DNA contamination and confidence estimation for single-cell genome data were assessed using ACDC software 2021–2022 [23]. Genome annotation was predicted using online databases, including the NCBI Prokaryotic Genome Annotation Pipeline (PGAP) [24], Clusters of Orthologous Groups (COG) (www.ncbi.nlm.nih.gov/research/cog-project), and Rapid Annotation using Subsystems Technology (RAST) (http://rast.nmpdr.org/rast.cgi). tRNA and rRNA genes were predicted using tRNAScan-SE [25] and RNAmmer, respectively [26]. The entire Genome Shotgun project has been deposited at DDBJ/ENA/GenBank under accession no. JAMBAR000000000.1. The version described in this paper is JAMBAR010000000. A circular map of the *Acinetobacter* sp. strain I-MWF genome was constructed using CGView [27].

A whole-genome-based taxonomic analysis of *A. guillouiae* I-MWF was performed using the Type Strain Genome Server (TYGS) [28]. The phylogenomic tree was constructed with FastME within the Genome BLAST Distance Phylogeny (GBDP) [29]. Pairwise genome comparisons were conducted using GBDP and inter-genomic distances [28]. Finally, trees were rooted at the midpoint, and branch supports were inferred from 100 pseudo-bootstrap replicates [30]. OrthoVenn2 [31] was used to identify orthologous proteins between the closest species, using the DIAMOND algorithm [32, 33]. Orthologous clusters were identified using the default parameters, with a 1e-5 e-value cut-off for all protein similarity comparisons.

The comparative genomic analysis was performed using the average nucleotide identity (ANI) calculated with the program JspeciesWS [34] for both best hits (one-way ANI) and reciprocal best hits (two-way ANI) between the genome sequence of *A. guillouiae* I-MWF and its closest relatives identified by whole-genome-based taxonomic analysis [34]. Typically, ANI values between genomes of the same species are above 95% [35].

To determine whether the *A. guillouiae* pangenome is open or closed, a comparative pangenome analysis was conducted. A total of 40 *Acinetobacter* genomes were initially considered, including 39 publicly available genomes retrieved from the NCBI database and the genome of our strain *A. guillouiae* I-MWF (GCF_025433935.1). Genome selection was restricted to assemblies annotated by NCBI RefSeq, and atypical genomes were excluded. After genome quality assessment, genomes showing evidence of contamination were removed, leaving 17 non-contaminated genomes for the final pangenome analysis. Pangenome analysis was performed using Roary v3.13.0 [36] with a BLASTp amino acid identity threshold of 90% for protein clustering. Roary output files were processed and visualized in R to characterize the core, accessory, and strain-specific genome fractions. Pangenome and core-genome rarefaction analyses were performed by examining changes in the cumulative number of gene clusters and core gene clusters as additional genomes were incorporated.

### Proteomic analysis

Extracellular proteins were recovered from *A. guillouiae* I-MWF cultures grown for 24 h in 50 mL of minimal salt medium (MSM) under different substrate conditions. The cultures were supplemented with: (i) 20 mg PET powder, (ii) 20 mg PET + 100 mg Tween 80, (iii) 20 mg PET + 200 mg Tween 80, or (iv) 500 mg Tween 80 as the sole carbon source. All cultivations were performed under identical conditions, and extracellular fractions were collected for proteomic analysis as described below.

#### In-solution digestion 

Proteins were digested in solution as follows: The pH of the samples was adjusted to 7.8 by adding ammonium bicarbonate to a final concentration of 100 mM. The proteins were reduced by the addition of DL-Dithiothreitol (DTT) (Sigma-Aldrich) to a final concentration of 5 mM and incubated at 37˚C for 30 min, followed by alkylation using iodoacetamide (IAA, Sigma-Aldrich) at a final concentration of 12 mM and incubated in the dark for 20 min. Sequencing-grade modified trypsin (Promega, Madison, WI, USA) was added to a concentration of 1:100 (trypsin: protein) before the samples were incubated at 37˚C for four hours, and then a second aliquot of trypsin was added to get a final concentration of 1:50 (trypsin: protein), and the samples were incubated at 37˚C overnight. The next day, formic acid (FA) was added to a final concentration of 0.5%. The peptides were cleaned up by C_18_ reversed-phase micro columns using an 2% Acetonitrile (ACN), 0.1% FA equilibration buffer and an 80% ACN, 0.1% FA elution buffer. The collected peptide samples were dried in a fume hood and resuspended in 15 µL 2% ACN, 0.1% FA before the LC-MS/MS analysis.

#### Mass spectrometry

Peptide samples were analysed by LC-MS/MS after injection onto an ultra-high-pressure nanoflow chromatography system (nanoElute, Bruker Daltonics). Peptides were loaded onto an Acclaim PepMap C_18_ trap column (5 mm, 300 μm id, 5 μm particle diameter, 100 Å pore size, Thermo Fisher Scientific) and separated on a Bruker Pepsep Ten C_18 analytical column_ (75 μm × 10 cm, 1.9 μm particle size, Bruker Daltonics). Mobile phase A (2% ACN, 0.1% FA) and mobile phase B (0.1% FA in ACN) were used to create a gradient over 45 min (from 2 to 17% B in 20 min, from 17 to 34% B in 10 min, from 34 to 95% B in 3 min, at 95% B for 12 min) at a flow rate of 400 nL/min and a column oven temperature of 50 °C. Peptides were analysed on a quadrupole time-of-flight mass spectrometer (timsTOF Pro, Bruker Daltonics) via a nanoelectrospray ion source (Captive Spray Source, Bruker Daltonics) in positive mode, controlled by the OtofControl 5.1 software (Bruker Daltonics). The ion transfer capillary temperature was 180 °C. A DDA method was used to select precursor ions for fragmentation, with one TIMS-MS scan and 10 PASEF MS/MS scans. The TIMS-MS scan was acquired between 0.60 and 1.6 V s/cm^2^ and 100–1700 m/z with a ramp time of 100 ms. The 10 PASEF scans contained a maximum of 10 MS/MS scans per PASEF scan at a collision energy of 10 eV. Precursors with a maximum of 5 charges, an intensity threshold of 5000 a.u., and a dynamic exclusion of 0.4 s were used.

Raw data from the LC-MS/MS were processed using Mascot Distiller (version 2.8.5) and searched using Mascot Daemon (version 2.8) against a database of Acinetobacteria protein sequences (720 entries) derived from the annotated genome of *A. guillouiae* I-MWF (described in the section above), using the following settings; precursor ion tolerance: 10 ppm, MS/MS fragment mass tolerance: 0.015 Da, protease: Trypsin, variable modifications: Carbamidomethyl (C) and Oxidation (M).

### Molecular cloning

Selected genes encoding lipases detected in the proteomic analysis were amplified by PCR and inserted into the expression vector pET21b, between the restriction sites *Nde*I and *Xho*I, in frame with the start codon of the vector and the encoding region of the histidine tag (HisTag x6) in the C-terminal. *Escherichia coli* BL21(DE3) and C43 strains were transformed by thermal shock. The recombinant cells were selected on LB agar medium using 100 µg/mL ampicillin as the selection factor [15]. The cultures were incubated at 37˚C overnight.

### Recombinant protein production

Recombinant *E. coli* strains were grown in LB broth supplemented with 100 µg/mL ampicillin. Cultures were incubated at 37 °C until an OD of 0.6-1 at 600 nm was reached. Recombinant protein expression was induced with 1 mM IPTG, and the temperature was then set to 20–22 °C for 12 h. Cell pellets were collected by centrifugation at 10,000 × *g* for 10 min. The pellets were resuspended in a buffer containing 100 mM Tris and 500 mM NaCl, adjusted to pH 7.4 with HCl, and sonicated to lyse the cells. Cell debris was removed from the supernatant by centrifugation at 38,000 × *g* for 20 min. The pellets were subjected to SDS-PAGE analysis. The purification step was performed according to Wagner-Egea et al. (2021) by applying the supernatant to Immobilised Metal Affinity Chromatography (IMAC).

### Molecular modelling

Extracellular hydrolases with potential to participate in PET depolymerisation were modelled. All annotated hydrolases from *A. guillouiae* I-MWF were subjected to signal peptide analysis using SignalP 6.0 [37], and only those predicted to be extracellular were selected. Subsequently, Phyre2 [38] was used for comparative modelling of the complete set of predicted extracellular hydrolases (after signal peptide removal), enabling identification of structurally related proteins and comparison with experimentally determined templates. AlphaFold 3 (https://alphafoldserver.com/) was also applied to the four lipases detected by proteomic analysis (MCT9977041, MCT9977977, MCT9977984 and MCT9979425) to obtain template-independent structural predictions, for subsequent structural comparison and docking analyses. Thereafter, docking of MCT9977041 and MCT9979425 with (MHET)_2_, and of MCT9980412 with four Tween-80 molecules, was performed using AutoDock Vina implemented in YASARA, as described previously [39]. The search space was centred on the predicted substrate-binding pocket, and the highest-scoring pose was selected for structural analysis. The reported binding energies correspond to YASARA docking scores (positive values indicate stronger binding) and were used only for qualitative comparison of predicted protein–ligand interactions.

Model analyses and figures were generated using Chimera 1.17.3 [40]. Molecular weights and isoelectric points (pI) of the proteins were computed with ProtParam on the ExPASy server [41].

## Results

### Strain identification

The isolated bacterial strain was identified as *A. guillouiae* through a comprehensive analysis of its complete 16 S rDNA gene sequence. The results establish 99.93% similarity between the isolated *A. guillouiae* strain I-MWF (MZ519889) and the reference *A. guillouiae* strain ATCC11171 (APOS01000028). *A. guillouiae* is a gram-negative, strictly aerobic bacterium, initially isolated from gasworks effluent [42]. Within the taxonomic hierarchy, it belongs to the Pseudomonadales Order and Moraxellaceae Family, comprising 108 species [43].

Furthermore, the investigation of phylogenetic relationships based on almost-complete 16 S rDNA gene sequences reveal that I-MWF (MZ519889) is most closely related to *A. guillouiae* strain ATCC 11,171 (APOS01000028), with 99.93% similarity (Figure S1). Additionally, a whole-genome-based phylogenetic tree constructed using TYGS consistently places strain I-MWF within the *A. guillouiae* species (ATCC 11171; APOS01000028).

To further support the previous results, we conducted an average nucleotide identity (ANI) analysis of closely related species, which indicates that the *A. guillouiae* I-MWF genome is 97% similar to that of *A. guillouiae* strain ATCC 11,171. Significantly, this ANI value surpasses the commonly accepted 95% cut-off for species delineation [28], thereby confirming that the I-MWF strain belongs to the *A. guillouiae* species.

### Isolation and growth of *A. guillouiae* I-MWF

The isolation strategy for *A. guillouiae* I-MWF consisted of colonisation of amorphous PET film, followed by cultivation in MSM medium containing PET powder. PET powder was prepared by solubilization and precipitation, giving a polymer with 11.3% crystallinity. The optimal temperature and pH were 23 °C and 7.5, but the strain could grow over a pH range of 7 to 8.

**Fig. 1:**
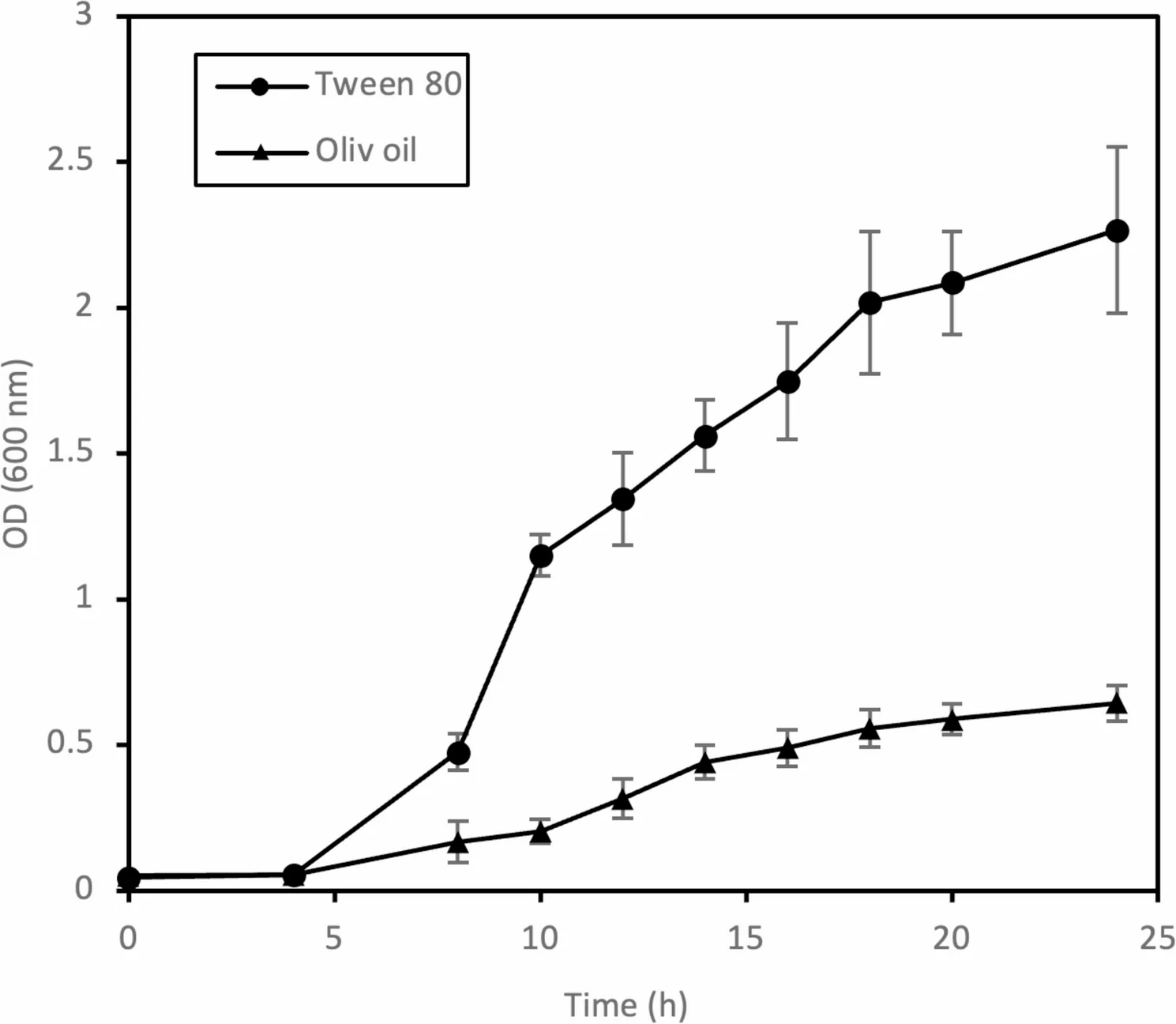
Growth curves of *A. guillouiae* I-MWF in MSM medium with Tween 80 and olive oil as the sources at 23 °C. The error bars represent standard deviations

The growth of *A. guillouiae* I-MWF on PET powder was compared with other carbon sources after 24 h. The highest growth was in Tween 80 and olive oil (Fig. 1) (Table 1), while minimal growth was detected in glucose. No growth was detected in ethyl terephthalate, terephthalic acid, ethylene glycol, or polysaccharides such as cellulose and chitin.

**Table 1:** Maximum cell density (OD_max_) and kinetic parameters (µ_max_ and t_g_) for *A. guillouiae* I-MWF cultured in MSM medium with different carbon sources for 24 h at 23˚C

Substrate	OD_max_	µ_max_ (h^− 1^)	t_g_ (h)
Tween 80	2.27 ± 0.29	0.52 ± 0.01	1.37 ± 0.04
Olive oil	0.63 ± 0.08	0.23 ± 0.04	3.26 ± 0.58

### PET active enzyme analyses

**Fig. 2:**
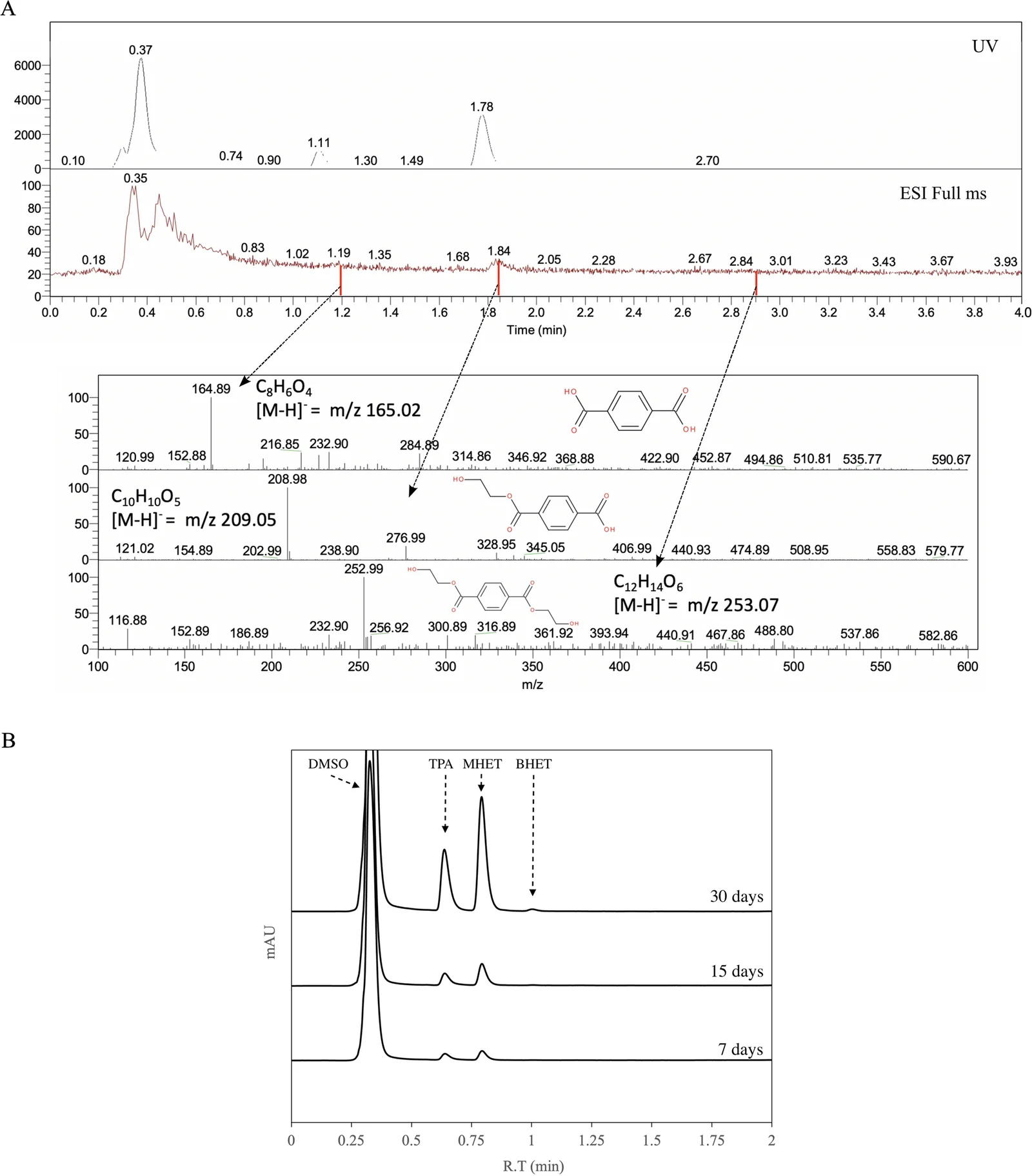
Analysis of PET depolymerisation products in the extracellular medium of *A. guillouiae* I-MWF. **A** LC-MS analysis. **B** HPLC-UV analysis

To investigate the production of extracellular PET-active enzymes during cultivation, culture supernatants collected after 7, 15, and 30 days were concentrated and incubated with PET powder for 72 h at 21–23 °C. The assay temperature was selected because it corresponds to the optimal growth temperature determined for *A. guillouiae* I-MWF and was intended to evaluate extracellular PET hydrolysis under physiologically relevant conditions for the strain. The resulting soluble products were analysed by LC-MS. TPA, MHET, and BHET were identified based on their retention times and mass spectra (Fig. 2A). PET-hydrolytic activity increased with cultivation time, as evidenced by the progressive accumulation of PET depolymerisation products in assays using extracellular fractions collected after 7, 15, and 30 days (Fig. 2B). TPA and MHET were detected in all samples, whereas BHET was only detected in the extracellular fraction collected after 30 days of cultivation, indicating increased PET depolymerisation capacity at later stages of growth.

**Fig. 3:**
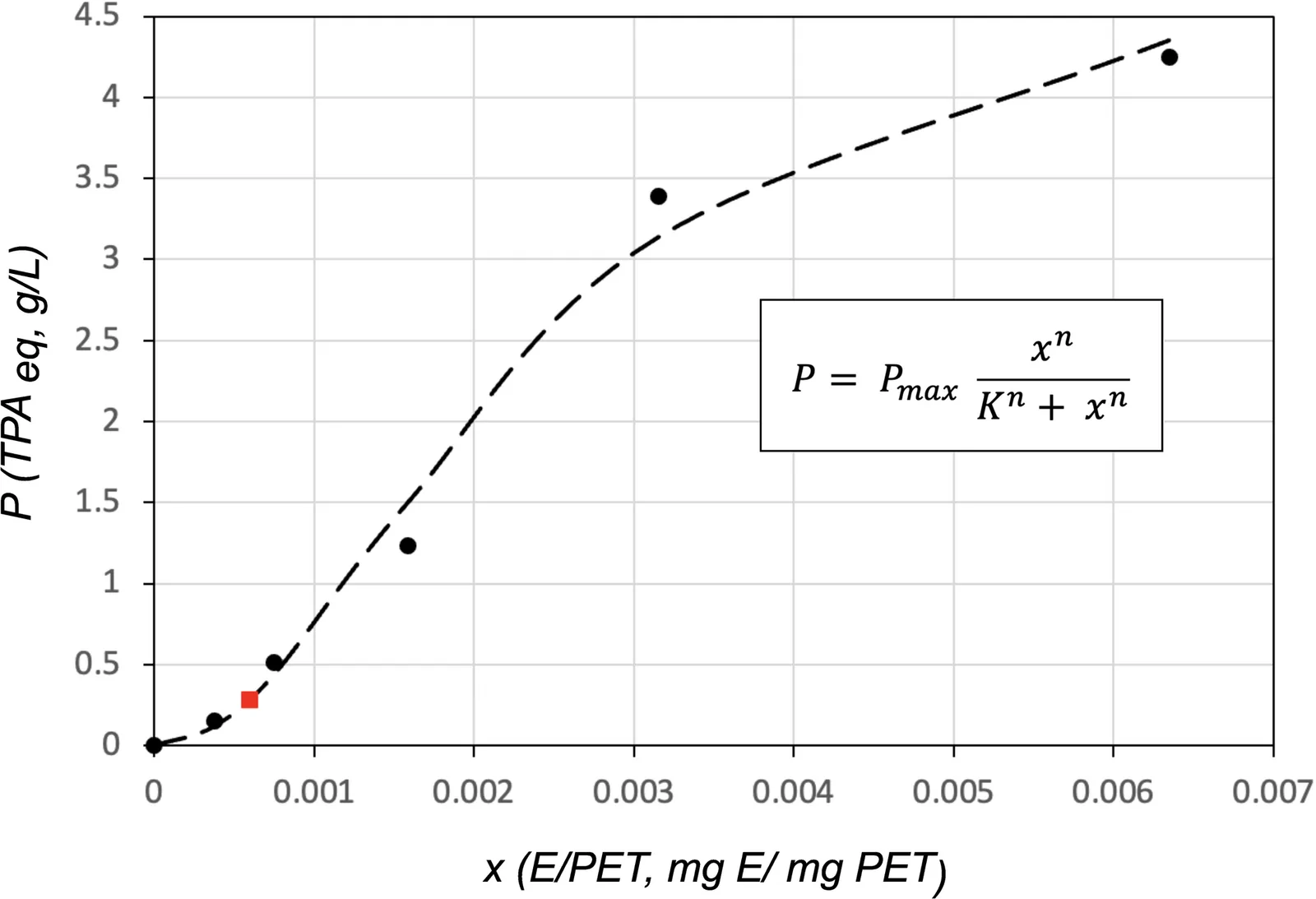
Estimation of the PET-hydrolytic activity of the extracellular extract from *A. guillouiae* I-MWF using an *Is*PETase standard curve. The extracellular extract collected after 30 days of cultivation (red dot) was compared with a calibration curve generated using purified *Is*PETase from *Ideonella sakaiensis*. The standard curve was fitted using a Hill-type saturation model ($\:{P}_{max}$=4.98 g/L, * K*=0.0024 g/g, *n*=2). Total soluble PET hydrolysis products released after 72 h incubation with 20 mg PET were quantified by HPLC and expressed as $\:{TPA}_{eq}$. The apparent PETase-equivalent activity of the extracellular extract was estimated by interpolation on the fitted curve

To quantitatively estimate PET-hydrolytic activity, the extracellular fraction collected after 30 days of cultivation was compared with a reference curve generated using purified *Is*PETase under identical assay conditions (Fig. 3). After 72 h incubation with PET, HPLC analysis detected 100.2 mg/L TPA, 183.6 mg/L MHET, and 2.4 mg/L BHET. The total concentration of soluble aromatic PET hydrolysis products was therefore estimated at 286.1 mg/L $\:{TPA}_{eq}$. Based on the Hill-model calibration curve, the PET-hydrolytic activity of the extracellular fraction corresponded to an apparent *Is*PETase-equivalent loading of 0.0006 mg enzyme per mg PET, equivalent to approximately 12 µg of purified *Is*PETase in the assay. Considering the total extracellular protein concentration (0.15 ± 0.02 mg/mL), the PET-hydrolytic activity represented approximately 8% of the activity expected for an equivalent mass of purified *Is*PETase.

### Genome features

**Fig. 4:**
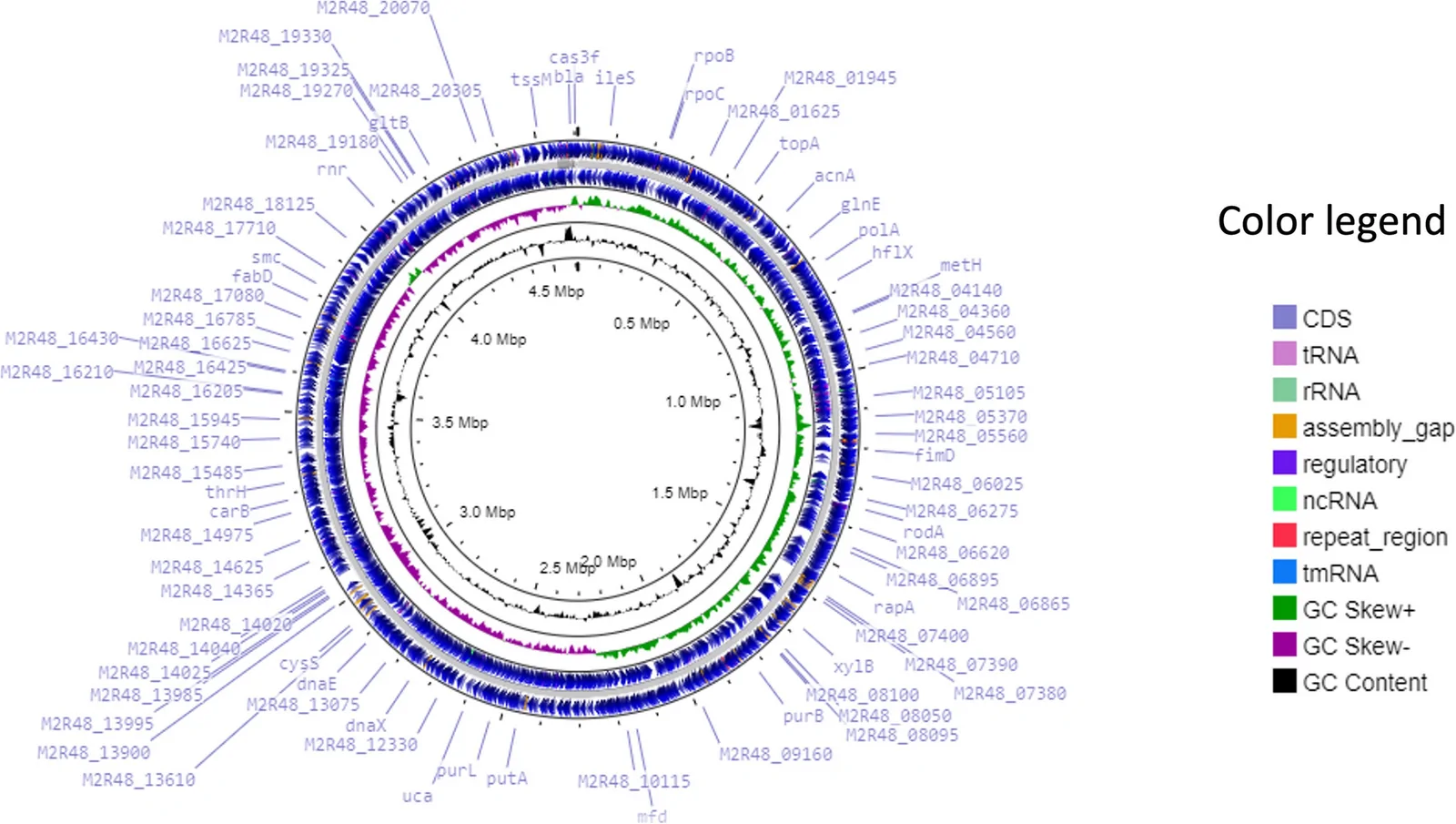
Graphical circular genome map of *A. guillouiae* I-MWF genome constructed using CGView (http://cgview.ca). The outermost ring shows the coding sequences with PGAP annotation (including tRNA, rRNA, tmRNA and ncRNA). The GC content is represented in black. The GC skew pattern is represented by the innermost ring, with purple indicating negative values and green indicating positive values

The *A. guillouiae* I-MWF genome comprises 87 scaffolds totalling 4.61 Mb, with an average GC content of 38.2% and an N50 of 4.6 Mb (Table 2). The NCBI Prokaryotic genome annotation pipeline (PGAP) identified a total of 4,303 genes, of which 4,178 were protein-coding, along with six rRNA genes (three 5 S, two 16 S, one 23 S) and 71 tRNA genes (Table 2; Fig. 4). In comparison, the genomes of other *A. guillouiae* strains (*n* = 16) deposited in the GenBank database range from 4.91 Mb (*A. guillouiae* NIPH 991; APPJ01) to 4.55 Mb (*A. guillouiae;* JAHPTS01) (Table 3).

**Table 2:** General features of the *A. guillouiae* I-MWF genome

Item	Value
Number of scaffolds	87
Total contig length (Mb)	4.61
G + C content (%)	38.2
Number of protein-coding genes	4,178
Number of predicted transfer RNAs	71
Number of predicted ribosomal RNAs	6
GenBank accession number	JAMBAR000000000.1

**Table 3:** General genomic features of *A. guillouiae* species deposited in GenBank database (as of August 2023)

Strain	Level	Size (Mb)	GC%	WGS	Scaffolds	CDS
CC160	Contig	4.64321	38.1	JAGIPP01	16	4123
SFC 500–1 A	Contig	4.81476	38.1	JAHWXT01	41	4346
BIGb0206	Contig	4.67876	38	JANUEF01	31	4291
WU_MDCI_Ag157	Contig	4.55717	38.1	JAHPTS01	49	4152
KCTC 23,200	Contig	4.84278	38.1	BBRY01	72	4486
UN03-107	Scaffold	4.70491	38.2	JAQEOA01	85	4292
UN03-29	Scaffold	4.69634	38.2	JAQEPJ01	88	4300
AM113-120	Scaffold	4.6844	38.2	JAQDVI01	102	4291
TUM15313	Contig	4.67043	38.2	BKPA01	182	4237
TUM15493	Contig	4.74103	38.2	BKVV01	284	4287
PgBE136	Chromosome	4.6231	38.1	Not available	1	4144
CCUG 2491	Contig	4.87842	38.1	VZOG01	78	4515
NBRC 110,550	Complete	4.64842	38.3	Not available	1	4164
NIPH 991	Scaffold	4.90577	38.2	APPJ01	4	4417
CIP 63.46	Scaffold	4.90228	38.2	APOS01	16	4479
MSP4-18	Contig	4.84896	38.1	ASQG01	94	4485

Based on sequence homology and coding domain sequences (CDSs), we categorised 3872 genes into COGs (Clusters of Orthologous Groups) [44], including putative or hypothetical genes and genes of unknown function, which mapped to 22 different COGs (Figure S2). The most abundant COG categories apart from the [S] function unknown (886 CDSs) and uncluttered COGs (570 CDS), were those assigned to [R] general function prediction (570 CDSs, 11.5%), [k] transcription (303 CDSs, 6.11%), [E] amino acid transport and metabolism (295 CDSs, 5.95%), [P] inorganic ion transport and metabolism (258, CDSs, 5.2%), [C] energy production and conversion (219, CDSs, 4.41%), [M] cell wall/membrane biogenesis (218, CDSs, 4.39%), [J] translation (205 CDSs, 4.13%), [I] Lipid transport and metabolism (205 CDSs, 4.13%).

**Fig. 5:**
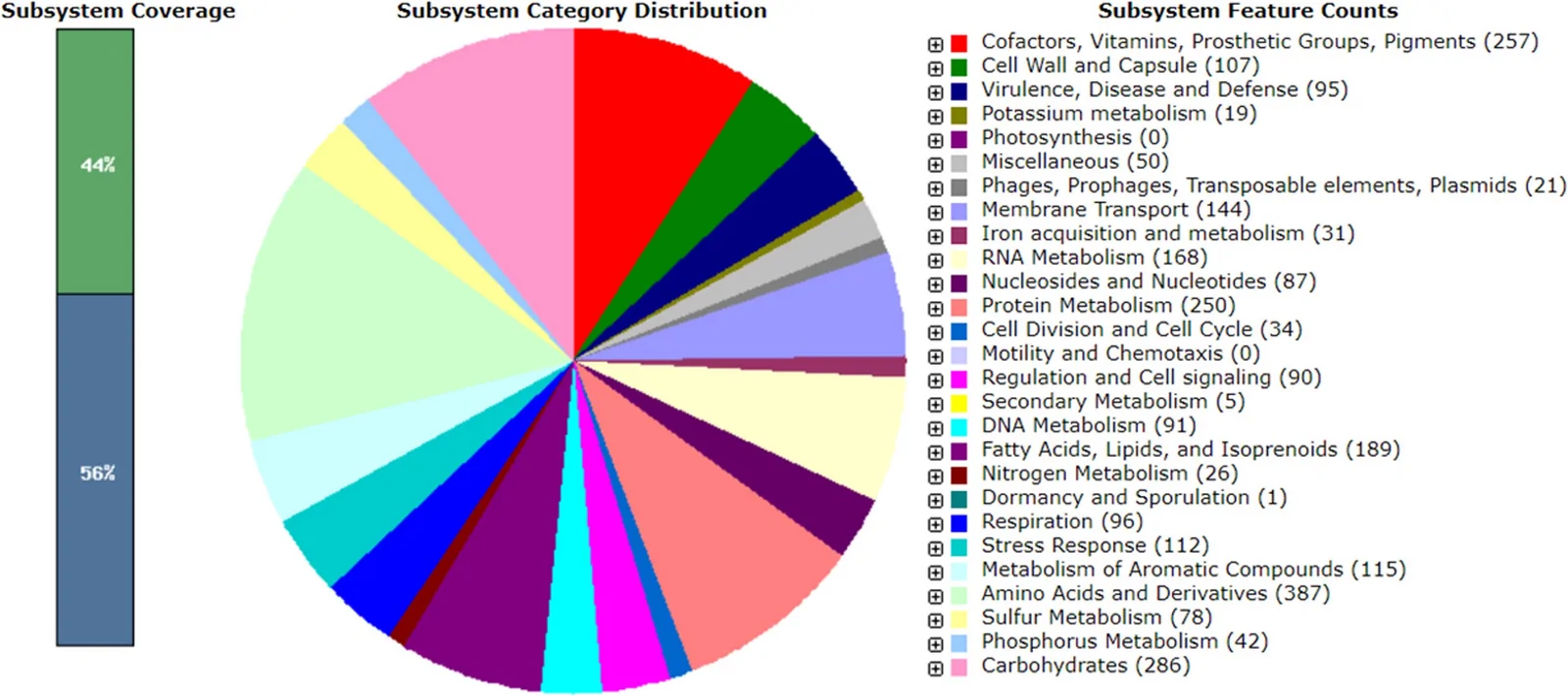
An overview of the subsystem categories assigned to the metabolic genes and pathways predicted in the genome of *A. guillouiae* I-MWF genome. The green part in the bar chart at the leftmost position corresponds to the percentage of proteins involved (44%), and the pie chart in the right panel demonstrates the fraction and count (parentheses in legend) of each subsystem feature

The subsystem distribution and general information on the potential functional distribution based on RAST annotation are illustrated in Fig. 5. Genes associated with amino acids and derivatives correspond to 387 ORFs, carbohydrates to 286 ORFs, cofactors, vitamins, prosthetic groups, and pigments to 257 ORFs, and protein metabolism to 250 ORFs. Fatty Acids, lipids, and isoprenoids (189 ORFs) were abundant among the SEED subsystem categories.

### Comparative genomic analysis

**Fig. 6:**
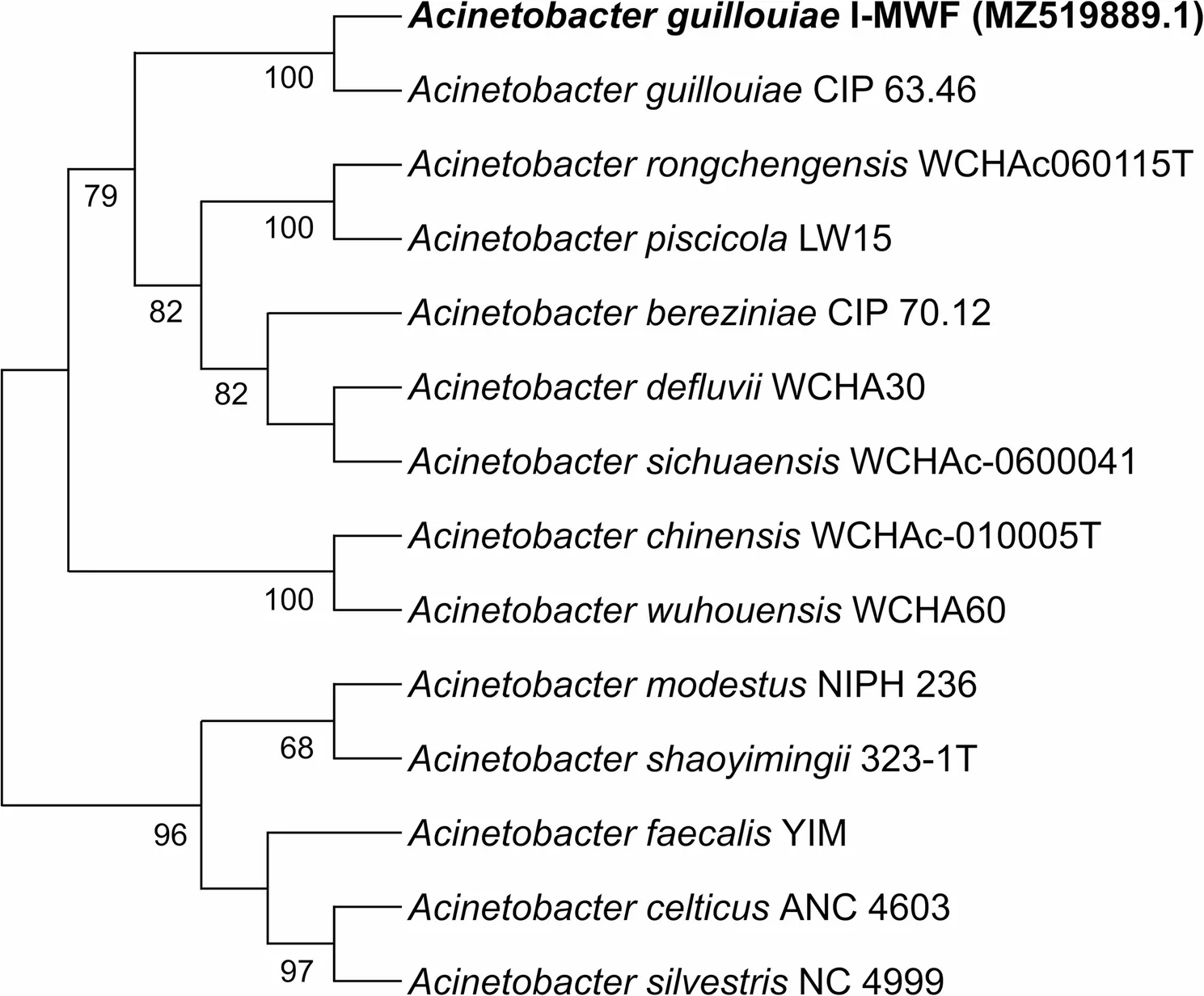
Phylogenomic tree based on genome sequences in the TYGS tree inferred with FastME 2.1.6.1 from Genome BLAST Distance Phylogeny approach (GBDP). The branch lengths are scaled in terms of the GBDP distance formula d5. The numbers above branches are GBDP pseudo-bootstrap support values > 50% from 100 replications. The tree was rooted at the midpoint

A whole-genome-based phylogenetic tree generated using the TYGS server showed that strain I-MWF is closely related to *A. guillouiae* CIP 63.46 (ATCC 11171) (APOS01000028), confirming the result of the 16 S rRNA-based analysis (Fig. 6). The ANI values between the I-MWF genome and all genomic sequences of *A. guillouiae* strains deposited in GenBank since 2023 are > 97%, exceeding the 95% cut-off for the same species, thereby confirming the assignment of the I-MWF strain to *A. guillouiae*.

Pangenome analysis of *A. guillouiae* I-MWF, compared with the closest species, *A. guillouiae* CIP 63.46 (ATCC 11171), was performed using orthoVenn2 (Figure S3). The pangenome comprised 3,678 clusters, including 65 orthologous clusters (with at least two copies in one genome) and 3,613 single-copy gene clusters. The core genome contained 3,629 proteins. Strain-specific clusters were unevenly distributed; the I-MWF strain showed 40 clusters potentially assigned to P: fatty acid biosynthetic process, P: cell adhesion, F: translation elongation factor activity, P: pathogenesis, P: protein secretion by the type V secretion system, F: uridine phosphorylase activity, F: protein serine/threonine kinase activity, and P: polysaccharide catabolic process. The remaining clusters are unannotated (with no Swiss-Prot Hit or Gene Ontology Annotation). The molecular functions assigned to all these clusters are transferase activity (GO:0016740) and nucleic acid binding (GO:0003676). For *A. guillouiae*, nine specific clusters were identified and assigned to P: translesion synthesis (GO:0019985), P: lipopolysaccharide biosynthetic process (GO:0009103), P: regulation of transcription (GO:0006355), and F: transferase activity (GO:0016757), while the other 5 clusters remained unannotated. The molecular function assigned to these clusters is transferase activity (GO:0016740). These results show that although the two strains belong to the same species, each genome encodes several unique features, possibly reflecting adaptation to the ecological niche.

**Fig. 7:**
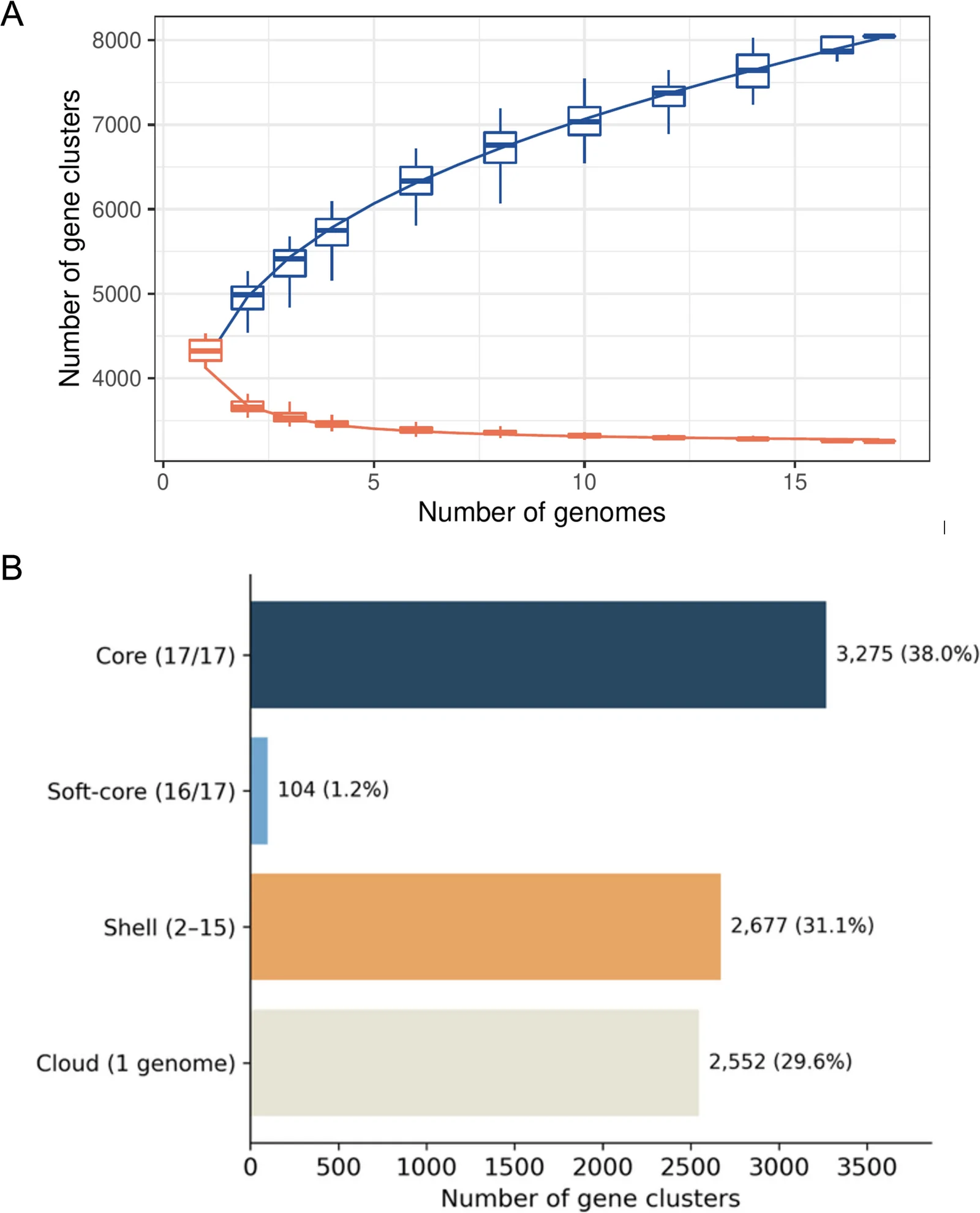
Pangenome structure and gene-cluster composition of *A. guillouiae* (*n* = 17 genomes). **A** Pangenome and core-genome rarefaction curves. The blue curve represents the cumulative pangenome size, which progressively increases with the addition of genomes, whereas the orange curve represents the core-genome size, which decreases and tends toward stabilisation. The absence of a clear plateau in the pangenome curve supports an open pangenome structure for *A. guillouiae*. **B** Pangenome composition based on 8,608 distinct gene clusters: core, present in all 17 genomes (3,275 clusters; 38.0%); soft-core, present in 16 of 17 genomes (104; 1.2%); shell, present in 2–15 genomes (2,677; 31.1%); and cloud, restricted to a single genome (2,552; 29.6%). The accessory genome (shell + cloud; 5,229 clusters; 60.7%) exceeds the core genome, consistent with the open pangenome indicated by the rarefaction analysis

To extend this pairwise comparison and assess the broader genomic diversity of *A. guillouiae*, a pangenome analysis was subsequently performed on the 17 genomes retained after quality filtering (Fig. 7A). The pangenome rarefaction curve showed a continuous increase in the cumulative number of gene clusters as additional genomes were included, without reaching a clear plateau. In contrast, the number of core gene clusters progressively decreased and tended towards stabilisation. These results indicate that *Acinetobacter guillouiae* exhibits an open pangenome, suggesting that additional gene families are expected to be identified as more genomes become available.

The gene presence/absence matrix further revealed substantial heterogeneity in the distribution of gene clusters across the 17 *A. guillouiae* genomes (Supplementary Excel Table). A total of 8,608 distinct gene clusters were identified, of which 3,275 were conserved across all 17 genomes and therefore constituted the strict core genome. In addition, 104 clusters were present in 16 of the 17 genomes, representing a highly conserved near-core fraction. In contrast, 2,677 clusters showed an intermediate distribution across 2–15 genomes, whereas 2,552 clusters were restricted to a single genome (Fig. 7B). The high proportion of variably distributed and strain-specific clusters highlights the extensive accessory genomic diversity of *A. guillouiae* and is consistent with the open pangenome structure inferred from the rarefaction analysis.

Within this variable gene repertoire, 244 gene clusters were detected exclusively in *A. guillouiae* I-MWF (GCA_025433935.1) and were absent from the other 16 genomes (Supplementary Excel Table). Most of these strain-specific clusters (203/244) encoded hypothetical proteins, whereas 41 were assigned putative functions. Among the most notable I-MWF-specific determinants was a complete CRISPR-associated module comprising cas1, cas3, csy1, csy2, csy3, and cas6f, suggesting a distinctive adaptive defence system in this strain. Additional strain-specific genes included the β-lactamase gene pse4 and the contact-dependent inhibition determinant cdiA, together with cdiI immunity genes, potentially contributing to interbacterial competition.

Genes associated with adhesion and cell-surface interactions, including ata, fimA, and ompA, were also identified within the I-MWF-specific repertoire. Moreover, several genes potentially involved in cell envelope and polysaccharide remodelling, including kfoC, capD, wbjC, wbpI, and eptC, were uniquely detected in I-MWF. The strain-specific fraction also contained genes associated with mobile genetic elements and genome rearrangement, including transposases, prophage integrases, XerC-family recombinases, and DNA helicases, pointing to a possible role for genome plasticity and horizontal gene acquisition in the differentiation of this strain.

Overall, the open pangenome structure observed at the species level, together with the large accessory gene pool and the 244 gene clusters specific to I-MWF, demonstrates substantial intraspecific genomic diversity within *A. guillouiae*. I-MWF is distinguished by an accessory gene repertoire associated with adaptive defence, interbacterial competition, adhesion, cell-surface and polysaccharide remodelling, and genome plasticity, which may contribute to its adaptation to its specific ecological niche.

### Putative enzymes involved in PET depolymerisation

The *A. guillouiae* I-MWF genome sequence allowed the annotation of at least 18 genes encoding extracellular hydrolases. The molecular weight (MW) and isoelectric point (pI) were theoretically obtained from the amino acid sequences (Table 4). The results revealed a wide range of MW, from 20,010 Da to 47,465 Da, with an average of around 34,000 Da. Similarly, pI values exhibited significant variability, ranging from 4.99 to 9.86, indicating adaptation to a broad pH spectrum. The diversity of these MW and pI values suggests that these extracellular proteins have diverse functions in bacterial physiology. In addition, certain proteins exhibited remarkable properties, such as the high pI of MCT9979697 and the high MW of MCT9979753.

**Table 4:** Molecular modelling of extracellular hydrolases selected from the annotated genome of *A. guillouiae* I-MWF (Confidence 100%*). MW are the molecular weights, and pI are the isoelectric points

1	GenBank Locus	MW (Da)	pI	Hit PDB template	Hit organism source	Hit info	Sequence identity (%)	Alignment coverage (%)
	MCT9976820	31,442.85	9.20	2gzs	Escherichia coli	Enterobactin Hydrolase	42	95
2	MCT9977041	32,349.69	7.75	1ex9	*Pseudomonas aeruginosa*	Lipase	48	95
3	MCT9977042	32,850.33	8.94	1ex9	*Pseudomonas aeruginosa*	Lipase	46	95
4	MCT9977607	35,302.48	6.35	4opm	*Acinetobacter baumannii*	Putative lipase	57	94
5	MCT9977974	44,832.75	5.64	3h2i	*Xanthomonas oryzae*	Esterase LipA	21	88
6	MCT9977975	42,493.07	5.56	2veo	*Candida antarctica*	Lipase A	19	98
7	MCT9977976	44,293.18	4.99	2veo	*Candida antarctica*	Lipase A	22	98
8	MCT9977977	40,913.09	5.83	2veo	*Candida antarctica*	Lipase A	20	98
9	MCT9977984	42,818.21	5.95	2veo	*Candida antarctica*	Lipase A	21	99
10	MCT9978617	31,185.54	6.91	6a6o	*Caldicellulosiruptor acetigenus*	Acetyl ester-xyloside bifunctional hydrolase	35	97
11	MCT9978941	35,636.54	8.94	4n5i	*Lacticaseibacillus rhamnosus*	Esterase B	25	97
12	MCT9979318	22,791.70	5.29	1uxo	*Bacillus subtilis*	Hydrolase	32	90
13	MCT9979425	32,709.89	5.87	1ex9	*Pseudomonas aeruginosa*	Lipase	46	95
14	MCT9979562	34,140.32	6.07	1ex9	*Pseudomonas aeruginosa*	Lipase	50	89
15	MCT9979616	34,210.89	6.63	2ek8	*Aneurinibacillus* sp.	Aminopeptidase	29	96
16	MCT9979697	20,010.21	9.86	4jgg	*Pseudomonas aeruginosa*	Lysophospholipase A	48	99
17	MCT9979753	47,465.09	9.16	2veo	*Candida antarctica*	Lipase A	19	95
18	MCT9979878	37,974.04	6.84	3gff	*Shewanella oneidensis*	IroE-like serine hydrolase	31	94

* Phyre2 confidence values correspond to the probability that the query sequence adopts the fold of the selected template and do not represent the accuracy of the final atomic model. Structural details, particularly in regions with low sequence identity, should therefore be interpreted with caution

The molecular structures of extracellular enzymes with potential ester hydrolase activity, modelled using the Phyre2 server, achieved a confidence level of 100% (Table 4, Figure S4). The Phyre2 confidence scores reflect fold-recognition confidence rather than structural accuracy; therefore, the resulting models were used primarily for comparative and hypothesis-generating analyses. The crystallographic structures used as templates showed alignment coverage ranging from 19% to 57%. Although no cutinase-like enzymes were identified, several putative lipases and esterases with potential relevance to the observed PET-hydrolytic activity were identified. All these structures share the typical α/β hydrolase fold.

The crystal structure of the lipase 1ex9 (PDB ID: 1ex9) from *Pseudomonas aeruginosa* has been the template for four lipases, with identities higher than 46% in coverage superior to 89%. The overall structures consist of a β-sheet of parallel strands surrounded by α-helices. All these lipases were modelled with open-form active sites. The lipase A (PDB ID: 2veo) from *Candida antarctica* has been the template for five lipases with identities ranging from 19 to 22% with covers higher than 95%.

The esterase LipA (PDB ID: 3h2i) from *Xanthomonas oryzae* has been the best template, although with a low identity (21%) in a coverage of 88%. Another esterase was predicted by using the esterase B (PDB ID: 4n5i) from *Lacticaseibacillus rhamnosus* as the best template, sharing 25% of identity in a cover of 97%. A third esterase was predicted based on the crystal structure (PDB ID: 4opm) of a putative lipase from *A. baumannii*, with an identity of 57% and a cover of 94%.

The extracellular predicted lipases and esterases from *A. guillouiae* can be grouped into five types. (1) Lipases similar to the 1ex9 lipase from *Pseudomonas aeruginosa* (MCT9977041, MCT9977042, MCT9979425, MCT9979562). (2) Lipases similar to the 2veo lipase from *Candida antarctica* (MCT9977975, MCT9977976, MCT9977977, MCT9977984, MCT9979753). Although these proteins share only 19–22% sequence identity with the template, the alignments cover 95–99% of the sequences and support assignment to the conserved α/β-hydrolase fold. Consequently, the models were used primarily to infer global structural organisation and catalytic motifs, whereas detailed active-site architectures and substrate interactions should be interpreted cautiously until experimentally validated. (3) Esterase similar to the 3h2i esterase from Xanthomonas oryzae (MCT9977974). (4) Esterase similar to the 4n5i esterase from *Lacticaseibacillus rhamnosus* (MCT9978941). (5) Lipase similar to the 4opm lipase from Acinetobacter baumannii (1 protein).

Although additional extracellular hydrolases were predicted, they seem to be active with substrates different to extracellular lipids or esters. Therefore, all these enzymes are unlikely to be active on PET. For example, MCT9976820, like the 4jgg lysophospholipase-A from *P. aeruginosa* is more likely active on the metabolism of membrane lysophospholipids than extracellular lipids; MCT9978617 is related to the 6a6o acetyl ester-xyloside from *Caldicellulosiruptor acetigenus*; MCT9979616 is similar to the 2ek8 aminopeptidase from *Aneurinibacillus* sp.; MCT9979878 is predicted as an IroE-like serine hydrolase, and MCT9976820 as a enterobactin hydrolase, showing high similarity to 3gff from *Shewanella oneidensis*, and 2gzs from *E. coli*, respectively. Finally, MCT9979318, like the 1uxo hydrolase from *Bacillus subtilis*, has an unknown function.

### Proteomics

The proteomic analysis of the extracellular fractions of *A. guillouiae* I-MWF cultivated with.

PET and Tween 80 has shown several hydrolases (Table 5). Four of these enzymes: MCT9979425, MCT9977041, MCT9977984, and MCT9977977, correspond to putative lipases previously listed in Table 4. The presence of signal peptides in these proteins indicates secretion and makes these proteins candidates for further investigation of the observed extracellular PET-hydrolytic activity, particularly under conditions supplemented with Tween 80. Recombinant expression of the selected lipases was evaluated in *E. coli* BL21(DE3) and C43. The expressed recombinant lipases were predominantly detected in the insoluble fraction, consistent with inclusion-body formation, whereas no clear recombinant protein band corresponding to MCT9977977 was detected under the conditions tested (Figure S5). Consequently, soluble recombinant enzymes suitable for biochemical characterisation could not be obtained.

Other proteins of interest identified may contribute indirectly to PET depolymerisation. These proteins include oxidative and detoxification enzymes, such as catalase (MCT9977107), glutathione peroxidase (MCT9980640), and alkyl hydroperoxide reductase (MCT9978043). The detection of these proteins indicates the presence of oxidative stress and detoxification responses under the tested cultivation conditions; however, their occurrence cannot be specifically attributed to PET exposure. All these enzymes lack a signal peptide, indicating intracellular localisation. A full table of detected proteins is available in Table S1.

**Table 5:** Hydrolases identified by proteomic analysis of *A. guillouiae* I-MWF exposed to PET and Tween 80

Protein		Locus	Signal Peptide	pI	MW [Da]	PET (20 mg)	Tween 80 + PET (100 mg)	Tween 80 + PET (200 mg)	Tween 80 (500 mg)
Triacylglycerol lipase		MCT9979425	pos. 21 and 22.	6.08	34,961		x	x	x
Triacylglycerol lipase		MCT9977041	pos. 21 and 22	7.75	34,554	x	x	x	x
Lipase family protein		MCT9977984	Lipoprotein signal peptide pos. 22 and 23	5.95	45,102	x	x	x	
Lipase family protein		MCT9977977	Lipoprotein signal peptide pos. 19 and 20.	5.83	42,869	x	x	x	x
Alpha/beta hydrolase		MCT9978322	no signal peptide	5.83	25,171	x	x		
Carboxy terminal-processing peptidase		MCT9977241	no signal peptide	7.15	80,383		x	x	
Amidohydrolase		MCT9978004	no signal peptide	8.35	40,440		x		
Bifunctional phosphoribosyl aminoimidazole carboxamide formyl transferase / IMP cyclohydrolase		MCT9979316	no signal peptide	5.50	56,021		x	x	
Catalase	MCT9977107		no signal peptide	6.07	57,287	x	x	x	
Glutathione peroxidase	MCT9980640		no signal peptide	4.59	20,133	x			
Alkyl hydroperoxide reductase subunit C	MCT9978043		no signal peptide	5.03	20,681	x		x	

### Molecular docking

**Fig. 8:**
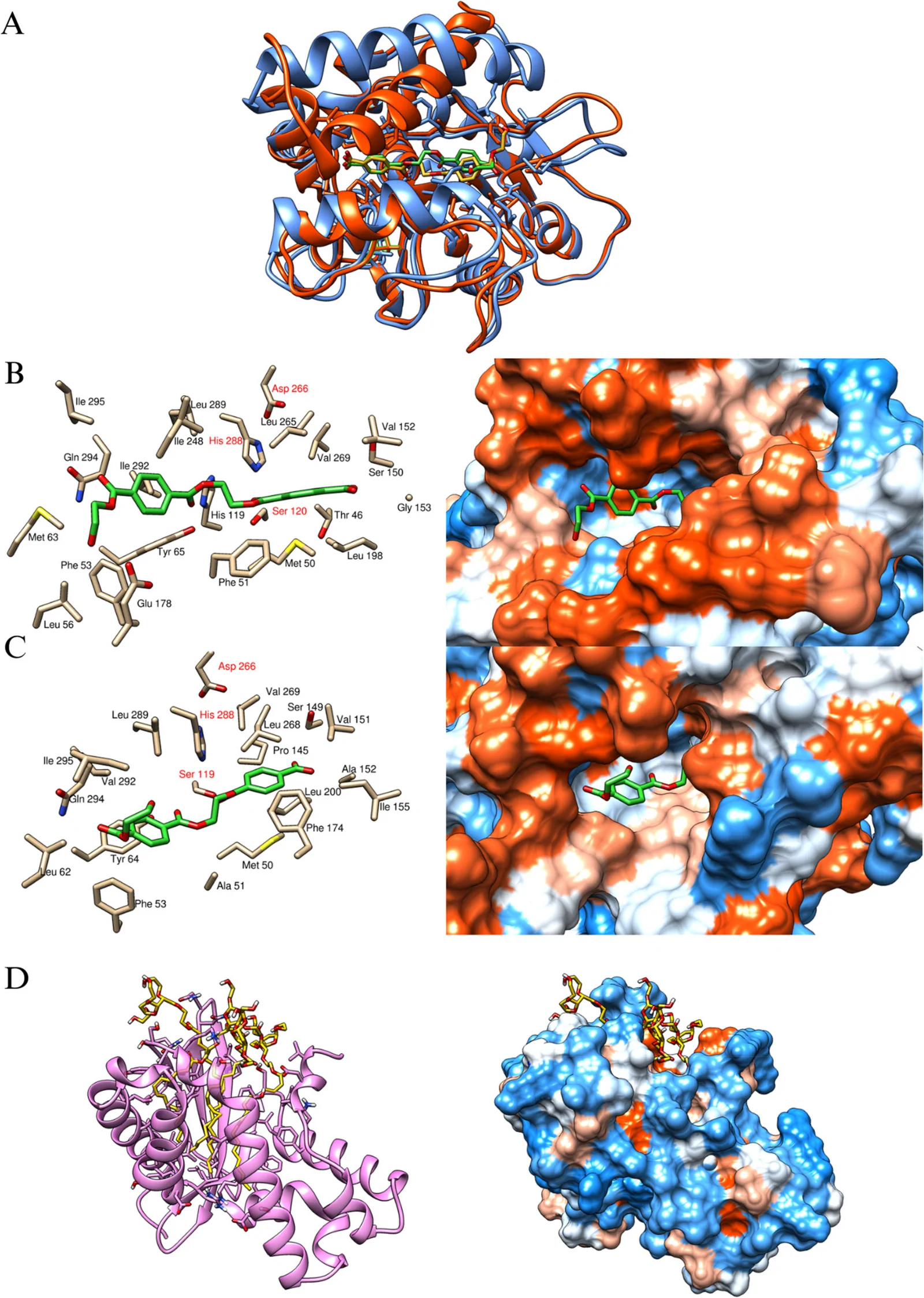
Comparative models and docking of the dominant lipases and the lipid transporter. **A** Structural superposition of MCT9979425 (blue) and MCT9977041 (orange) modelled (with AlphaFold 3, Figure S6) in complex with MHET_2_. The most pronounced backbone difference is at α-helix 6. **B** Catalytic site of MCT9979425. Residues within the binding pocket are shown as sticks; the catalytic triad (Ser120–His288–Asp266) is labelled in red. Right, molecular surface coloured by hydrophobicity (red, more hydrophobic; blue, more hydrophilic). **C** Catalytic site of MCT9977041. Pocket residues as sticks; catalytic triad (Ser119–His288–Asp266) labelled in red. Right, corresponding hydrophobicity surface (red to blue as in panel B). **D** Model of MCT9980412 complexed with four molecules of Tween-80 (yellow sticks). Right, surface hydrophobicity rendering as above

Proteomic profiling of *A. guillouiae* cultivated on PET or on PET supplemented with Tween-80 identified two lipases, MCT9979425 and MCT9977041, as dominant enzymes with potential activity against PET. To explore substrate recognition, we docked MHET2 into both lipases. Superposition of the resulting complexes (Fig. 8A) highlights α-helix 6 as the principal structural divergence between the models, a difference that is likely to influence access to the catalytic pocket. In addition, a lipid transporter protein was docked with four Tween-80 molecules. The predicted complexes showed Yasara docking scores of 8.41 and 8.16 kcal/mol for MCT9977041 and MCT9979425 with PET oligomers, respectively, whereas docking Tween-80 to the transporter MCT9980412 yielded a score of 4.70 kcal/mol. These values should be interpreted qualitatively because the structures are computational models and have not been experimentally validated.

At the active site, the canonical catalytic triad is conserved, comprising Ser119–His288–Asp266 in MCT9977041 and Ser120–His288–Asp266 in MCT9979425. Most pocket residues are identical or conservatively substituted; notable differences include Gly153 (MCT9979425) versus Ala152 (MCT9977041), Phe51 (MCT9979425) versus Ala51 (MCT9977041), and Thr146 (MCT9979425) versus Pro145 (MCT9977041). Conversely, Ile155 and Phe174 are unique to MCT9977041 (with Phe174 residing on α-helix 6), whereas Leu56, His119, Ile248 and Glu178 are unique to MCT9979425 (with Glu178 on α-helix 6). MCT9979425 is also slightly longer overall than MCT9977041, as reflected in their molecular weights (Table 4).

In both models (Fig. 8B, C), the catalytic pocket is partially occluded, consistent with the well-known lipase lid mechanism, in which a hydrophobic cap modulates substrate access. Differences in the composition and positioning of α-helix 6, including a higher local frequency of aromatic residues in MCT9979425, likely tune lid dynamics and, by extension, active-site accessibility. These sequence and structural features may therefore underlie the distinct docking poses observed for MHET2 and could translate into different catalytic preferences in vivo. We emphasise that these are in silico predictions that warrant experimental validation in future work.

For cultures grown on Tween 80 as the sole carbon source, the lipid transporter protein MCT9980412 was detected. Docking with four Tween-80 molecules (Fig. 8D) yielded a stable complex, consistent with a multivalent hydrophobic binding surface and providing a plausible molecular rationale for the robust growth observed with Tween 80 alone.

## Discussion

Accordingly, the activity observed here should be interpreted as partial extracellular PET depolymerisation rather than complete PET biodegradation, as assimilation or mineralisation of PET-derived monomers was not demonstrated.

Sludge is a source of inorganic and organic nutrients that support microbial growth, including microorganisms with the potential to degrade PET [45]. In the present work, *A. guillouiae* I-MWF was successfully isolated by introducing amorphous PET film into sludge samples to select for bacteria capable of colonising the plastic, followed by isolation of the strain in enrichment cultures. For this strategy, we hypothesised that film-colonising bacteria could develop the ability to depolymerise PET. In addition, amorphous PET films may be more susceptible to bacterial attack than highly crystalline ones, thereby facilitating colonisation.

*A. guillouiae* I-MWF was initially identified through phylogenetic analysis of the complete 16 S ribosomal gene. Phylogenomic and comparative genomic analyses have confirmed the species. The 16 S phylogeny indicates that *A. guillouiae* I-MWF is closely related to *A. bereziniae*. Genomic analysis shows that the strain *A. guillouiae* CIP 63.46 is the closest to *A. guillouiae* I-MWF, although the two strains possess unique features linked to ecological niche adaptations. *A. guillouiae* strains are mainly found in sludge. However, recently, an *A. guillouiae* strain capable of degrading polyethylene through alkane hydroxylation and alcohol dehydrogenation was isolated from insect larvae [46].

The growth of *A. guillouiae* I-MWF in Tween 80 and olive oil demonstrates its ability to utilise lipids under optimal cultivation conditions. In contrast, it could not grow on carbohydrates such as cellulose and chitin and showed only minimal growth on glucose. Although *A. guillouiae* I-MWF partially degrades PET powder, no depolymerisation products were detected when non-pre-treated PET, such as pieces of drink bottles, was treated with this bacterium.

Growth of *A. guillouiae* I-MWF on terephthalic acid (TPA), the aromatic monomer of PET used as the sole carbon source, was not observed. Computational studies indicate that TPA at neutral pH is unlikely to cross the cell membrane by diffusion [47]. Although genes encoding a benzoate/H+/H+ (benE) symporter and a major facilitator superfamily (MFS) transporter are present, no putative TPA transporter gene was identified in the *A. guillouiae* I-MWF genome. However, TPA transporters are poorly characterised. Despite this, *A. guillouiae* I-MWF has putative genes involved in the protocatechuate acid pathway, which is linked to the metabolism of phenolic compounds. The growth of *A. guillouiae* I-MWF on the other PET monomer, ethylene glycol, was also undetectable. Thus, together with the substantially stronger growth observed on Tween 80 and olive oil, these results indicate that I-MWF is primarily a lipolytic bacterium possessing secondary extracellular PET-hydrolytic activity, rather than a bacterium metabolically adapted to PET utilisation.

The extracellular PET-hydrolytic activity was evaluated at 23 °C, the optimal growth temperature of I-MWF. Consequently, the present experiments were designed to assess activity under physiologically relevant conditions rather than to determine the temperature optimum of the secreted hydrolases. Temperature-dependent characterisation will require purified active enzymes. Partial PET depolymerisation is supported by the detection of depolymerisation products in HPLC and LC-MS analyses of PET powder treated with extracellular enzymes from *A. guillouiae* I-MWF. The main depolymerisation product was MHET. Similar results were reported for *Is*PETase from *Ideonella sakaiensis* [11], marine PET2 [15], and leaf-branch compost cutinase (LCC) from a bacterial origin [12]. In addition, the extracellular enzymatic extract from *Stenotrophomonas maltophilia* also yielded MHET as the major depolymerisation product [48]. By contrast, the *Candida antarctica* lipase (CaL) produced mainly BHET [49]. However, the product ratio profile may vary with factors such as incubation time, temperature, and the enzyme-to-polymer ratio [14].

To provide a quantitative context for the observed PET depolymerisation, the activity of the extracellular fraction was compared with a calibration curve generated using purified *Is*PETase under identical assay conditions. Because product formation approached a plateau at higher enzyme-to-PET ratios, a Hill-type saturation model was empirically used to fit the calibration data, enabling interpolation across the entire concentration range without assuming a linear response. Based on this analysis, the extracellular extract corresponded to an apparent PETase-equivalent loading of approximately 12 µg *Is*PETase and represented about 8% of the activity expected for an equivalent mass of purified enzyme. Although this activity is considerably lower than that of *Is*PETase, the extracellular fraction consisted of a complex protein mixture rather than a purified PET hydrolase. These results therefore support the presence of extracellular PET-active enzymes while suggesting that PET hydrolysis is a secondary activity rather than the primary physiological role of the secreted enzyme repertoire. Total extracellular protein concentration was not monitored at each sampling time; therefore, the time-dependent accumulation of PET hydrolysis products cannot be directly related to changes in extracellular enzyme abundance. The temporal profiles should consequently be interpreted as cumulative product formation rather than as changes in specific enzymatic activity.

In the genome of *A. guillouiae*, I-MWF, we identified at least 18 genes encoding extracellular hydrolases. However, the genome lacks cutinase-like enzymes but is rich in putative lipases and esterases. Indeed, these enzymes catalyse the hydrolysis of ester bonds, but they have different substrate specificities and functions. Analysis of 17 *A. guillouiae* genomes indicated an open pangenome and substantial intraspecific genomic diversity. The large accessory component suggests considerable strain-level variation in metabolic and adaptive potential. However, whether the extracellular hydrolases identified in I-MWF represent strain-specific adaptations or are broadly distributed within *A. guillouiae* requires further comparative genomic investigation.

Proteomic analysis provided further insight into extracellular enzymatic processes that may contribute to PET depolymerisation. Four distinct lipases were identified in the extracellular fractions. Lipases MCT9979425 and MCT9977041 are similar to a lipase from *P. aeruginosa*, while lipases MCT9977977 and MCT9977984 are analogous to lipase A, CalA (2veo) from *C. antarctica*. Of particular interest are lipase MCT9979425 and MCT9977041, as they are the most prevalent hydrolases in the samples. Although proteomic analysis identified several extracellular lipases, the present data do not permit assignment of the observed PET-hydrolytic activity to any individual enzyme. Direct biochemical characterisation of purified active proteins will be required to establish their contribution to PET depolymerisation.

Proteomic analysis also detected proteins associated with oxidative-stress defence, including catalase, glutathione peroxidase and alkyl hydroperoxide reductase. Their presence may reflect cellular responses to oxidative or metabolic stress under the cultivation conditions used. However, the current experimental design does not establish that these proteins were specifically induced by PET or its hydrolysis products, and their detection should therefore not be interpreted as evidence of an oxidative mechanism of PET depolymerisation. Comparative quantitative proteomic analyses under controlled PET, TPA and carbon-source conditions would be required to establish such a relationship.

The molecular models obtained correspond to extracellular lipases and esterases predicted from the genome analysis of *A. guillouiae* I-MWF. All share the typical α/β-hydrolase fold. They vary in molecular weight (20–47 kDa) and pI, suggesting distinct functions. The catalytic triad is well conserved across all models. Differences in the position and composition of residues in the sixth α-helical segment (α6) of lipases MCT9979425 and MCT9977041 may contribute to their enzymatic activity. Notably, lipase MCT9977041 appears to adopt a more closed conformation, potentially limiting substrate access to the catalytic site. Previous studies on the lipase from *P. aeruginosa* (PAL) have demonstrated that α-helical segment 6 plays an important role in shaping the active-site conformation [50]. Given the structural similarity between PAL and the two lipases analysed here, differences in α-helical segment 6 could indeed affect enzymatic activity, especially considering that a more open active site is generally associated with higher catalytic performance.

Despite the structural implications of the lid, several lipases have been reported to be active against PET. For example, the highly promiscuous lipase B from *Candida antarctica* uses triacylglycerides as natural substrates but can also hydrolyse polyesters, including PET oligomers [51]. Lipases from *Pseudomonas chlororaphis* have shown hydrolytic activity on petroleum-based polymers, such as poly-(ε-caprolactone) (PCL) and polyethylene succinate (PES) [52]. A comparative study has shown that a lipase from *Thermomyces lanuginosus* produced slightly higher amounts of larger fragments from PET than the cutinases from *Thermobifida fusca* and *Fusarium solani* [53]. *Acinetobacter* is a genus characterised by a wide variety of genes encoding lipases. Indeed, the *A. guillouiae* I-MWF genome encodes a variety of extracellular lipases and esterases that could potentially be involved in the partial depolymerisation of PET powder.

It is important to emphasise that these conclusions are based on predicted structural models. Therefore, it would be valuable to obtain experimentally determined 3D structures and perform enzymatic activity assays for MCT9979425 and MCT9977041 to better understand how differences in residues and conformations influence their function. Although these enzymes were produced in recombinant *E. coli* strains, they precipitated as inclusion bodies, and their activity could not be determined. Producing active recombinant lipases in *E. coli* is typically challenging, and a combination of cultivation optimisation, fusion tags, and co-expression of chaperones could improve the soluble expression of recombinant lipases in future studies.

In addition to lipases, proteomic analysis identified other proteins of interest. Notably, the ABC transporter MCT9980412 was the most abundant protein in samples grown on Tween 80 as the sole carbon source. This transporter is likely involved in lipid uptake, and docking analysis suggested a favourable interaction with Tween 80, consistent with the observed growth of *A. guillouiae* I-MWF on this substrate.

Consistent with these observations, *A. guillouiae* I-MWF secretes lipases associated with partial hydrolysis of amorphous PET. This activity suggests a potential role in the initial transformation of PET, leading to the formation of depolymerisation products. However, the ecological relevance of this process remains to be demonstrated. The observed co-expression of oxidative- and detoxification-related proteins may reflect a general stress response to PET or its transformation products, although the specific roles of these proteins in PET-associated processes require further investigation.

## Conclusions

*Acinetobacter guillouiae* I-MWF is a lipolytic, sludge-derived bacterium capable of partially depolymerising low-crystallinity PET via extracellular enzymes. Although the strain does not assimilate PET or its monomers, its secretion of lipases and production of PET-derived intermediates indicate that lipolytic bacteria may contribute to partial PET depolymerisation. Genomic and proteomic analyses reveal a diverse repertoire of extracellular hydrolases, identifying candidate enzymes that may drive this process. Together, these findings expand current understanding of microbial interactions with synthetic polymers and suggest that lipolytic bacteria may possess side activities capable of partial PET depolymerisation under laboratory conditions.

## Supplementary Information

Supplementary Material 1.

Supplementary Material 2.

## Data Availability

The entire Genome Shotgun project has been deposited at DDBJ/ENA/GenBank under the accession no. JAMBAR000000000.1. The version described in this paper is JAMBAR010000000.

## Abbreviations

ACN: Acetonitrile
ANI: Average nucleotide identity
BHET: Bis(2-hydroxyethyl) terephthalate
BCA: Bicinchoninic acid
CID: Collision-induced dissociation
COG: Clusters of orthologous groups
DDA: Data-dependent acquisition
DDBJ: DNA data bank of Japan
DTT: Dithiothreitol
FA: Formic acid
FT-IR: Fourier-transform infrared spectroscopy
GBDP: Genome BLAST distance phylogeny
GC: Guanine-cytosine
HESI: Heated electrospray ionization
HisTag: Histidine tag
HPLC: High-performance liquid chromatography
IAA: Iodoacetamide
IMAC: Immobilized metal affinity chromatography
LB: Lysogeny broth
LCC: Leaf-branch compost cutinase
LC-MS: Liquid chromatography–mass spectrometry
MHET: Mono(2-hydroxyethyl) terephthalate
MFS: Major facilitator superfamily
MSM: Minimal salt medium
ncRNA: Non-coding RNA
ORF: Open reading frame
PASEF: Parallel accumulation–serial fragmentation
PCR: Polymerase chain reaction
PET: Polyethylene terephthalate
PGAP: Prokaryotic genome annotation pipeline
RAST: Rapid annotation using subsystems technology
rRNA: Ribosomal ribonucleic acid
SDS: Sodium dodecyl sulfate
TPA: Terephthalic acid
TYGS: Type strain genome server
