# Supplementary material for: Acinetobacter guillouiae, a lipolytic strain isolated from sludge capable of partially depolymerising polyethylene terephthalate: genomic, proteomic, and biochemical insights
Source: BMC Microbiol. 2026 Aug 26;26:763. doi: 10.1186/s12866-026-05567-7 (PMC13520237; doi:10.1186/s12866-026-05567-7)
Supplement: Supplementary file 1 [file 12866_2026_5567_MOESM1_ESM.pdf]

Supplementary material

Acinetobacter guillouiae, a Lipolytic Strain Isolated from Sludge Capable of Partially Degrading Polyethylene Terephthalate: Genomic, Proteomic, and Biochemical Insights

Naheed Akhtar[1,2], Afef Najjari[3], Anna Magnone[1], Esma Ceylan Yerebakan[4], Katja Bernfur[5], Cecilia Tullberg[1], Muhammad Siddique Awan[2], Zahid Majeed[6], Carl Grey[1], Baozhong Zhang[7], Javier A. Linares-Pastén[1]*

[1]Division of Biotechnology and Applied Microbiology, Department of Process and Life Siences Engineering, Faculty of Engineering (LTH), Lund University, P.O. Box 124, SE-22100 Lund, Sweden.

[2]Department of Zoology, The University of Azad Jammu and Kashmir, Muzaffarabad, Pakistan

[3]Université Tunis El Manar, Faculté des Sciences de Tunis, LR03ES03 Laboratoire de Microbiologie et Biomolécules Actives, 2092, Tunis, Tunisie.

[4] Department of Biology, Faculty of Sciences, Karadeniz Technical University, 61080 Trabzon, Turkey

[5] Division for Biochemistry and Structural Biology, Department of Chemistry, Lund University, P.O. Box 124, SE-22100 Lund, Sweden

[6] Department of Biotechnology, The University of Azad Jammu and Kashmir, Muzaffarabad, Pakistan

[7]Centre for Analysis and Synthesis, Department of Chemistry, Faculty of Engineering (LTH), Lund University, P.O. Box 124, SE-22100 Lund, Sweden.

* Correspondence: javier.linares-pasten@ple.lth.se

Figure S1. Phylogenetic tree of *A. guillouiae* I-MWF and its closest related species based on 16S rRNA sequences. The evolutionary history was inferred using the neighbour-joining method. The percentage of replicate trees in which the associated taxa clustered together in the bootstrap test (1000 replicates) is shown next to the branches. The tree was rooted with *P. aeruginosa* DSM (NR 026078.1). Evolutionary analyses were conducted in MEGAX v10.2.6. GenBank accession numbers of sequences are shown in parentheses.

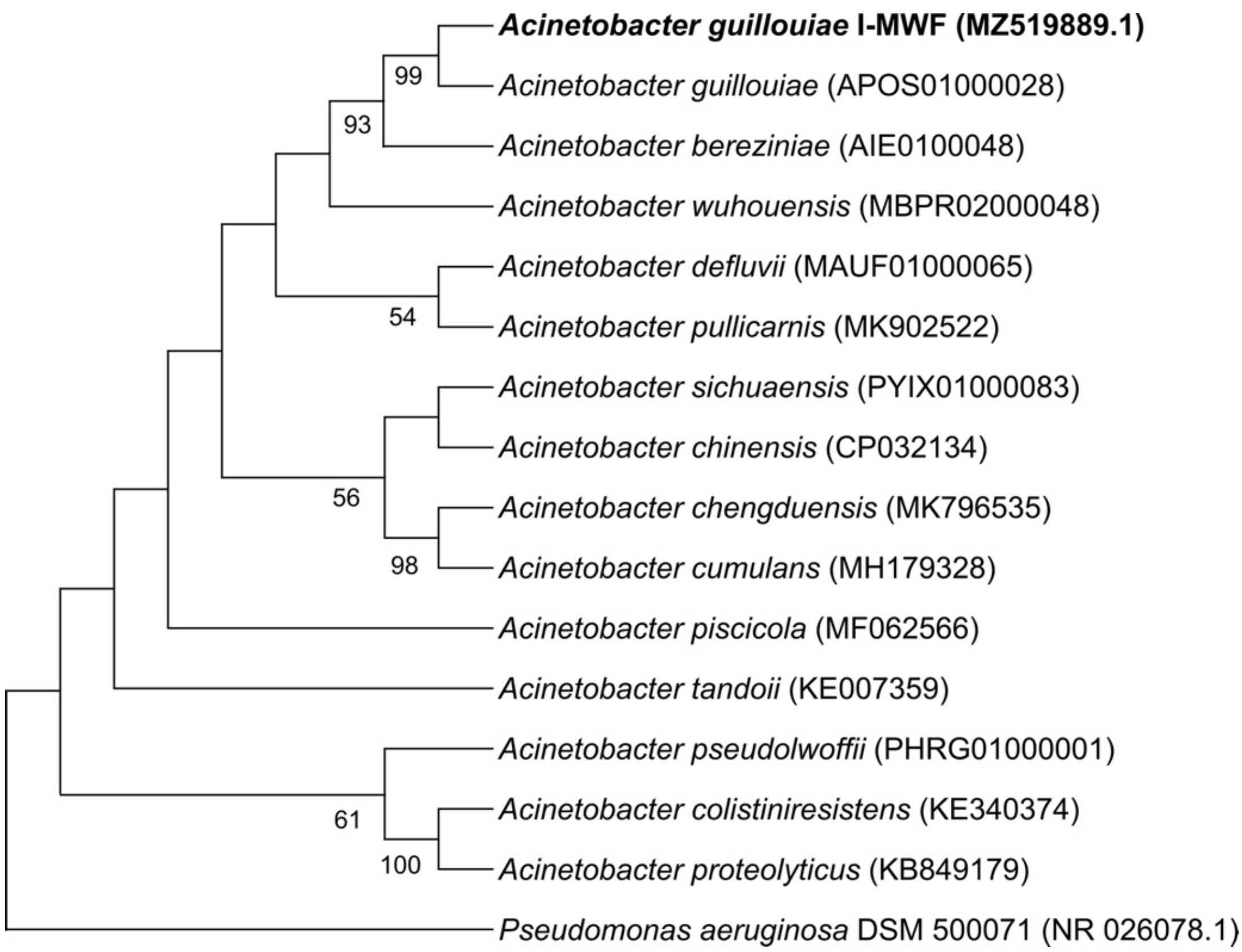

Figure S2. COG functional classification of *A. guillouiae* I-MWF genome. The X-axis represents the number of genes; the Y-axis is the COG function class.

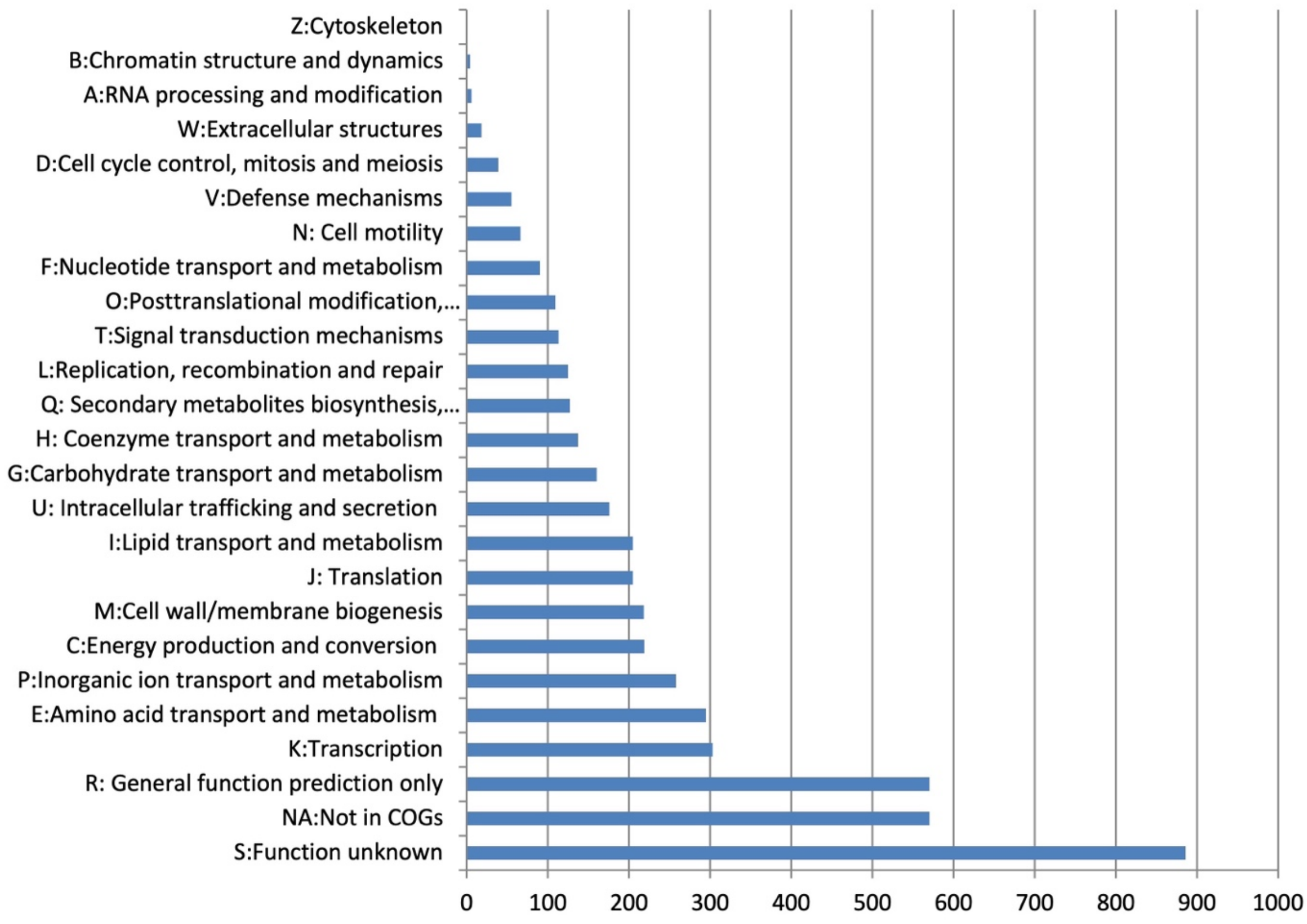

Figure S3. Venn diagram showing the distribution of shared and unique orthologous gene clusters among A. guillouiae I-MWF and A. guillouiae CIP 63.46 strains as visualised by OrthoVenn2. A total of 3629 shared clusters of orthologous groups were identified in these 2 strains.

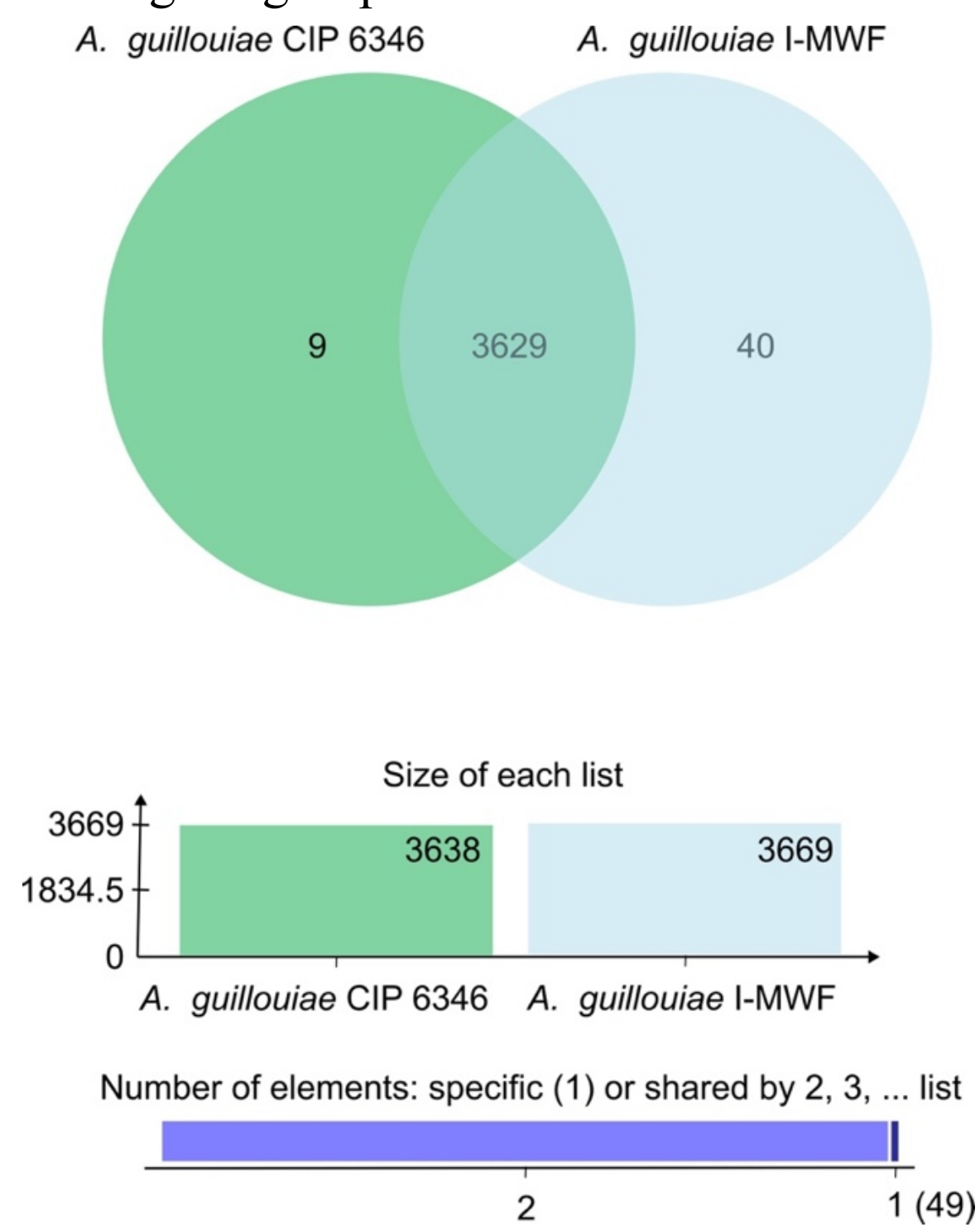

Figure S4. Molecular models of representative extracellular putative lipases and esterases from *A. guillouiae* I-MWF. (A) Overlapped models based on lipase 1xe9 from *Pseudomonas aeruginosa*. Docking of MCT9977042 with $(MHET)_4$, the ligand is shown in green. (B) Overlapped models based on lipase A 2veo from *Candida antarctica*. (C) Model-based in esterase LipA 3h2i from *Xanthomonas oryzae*. (D). Model-based in esterase B 4n5i form *Lacticaseibacillus rhamnosus*. (E) Model-based on a putative lipase 4opm from *Acinetobacter baumannii*. In all models, the predicted catalytic triad is in yellow.

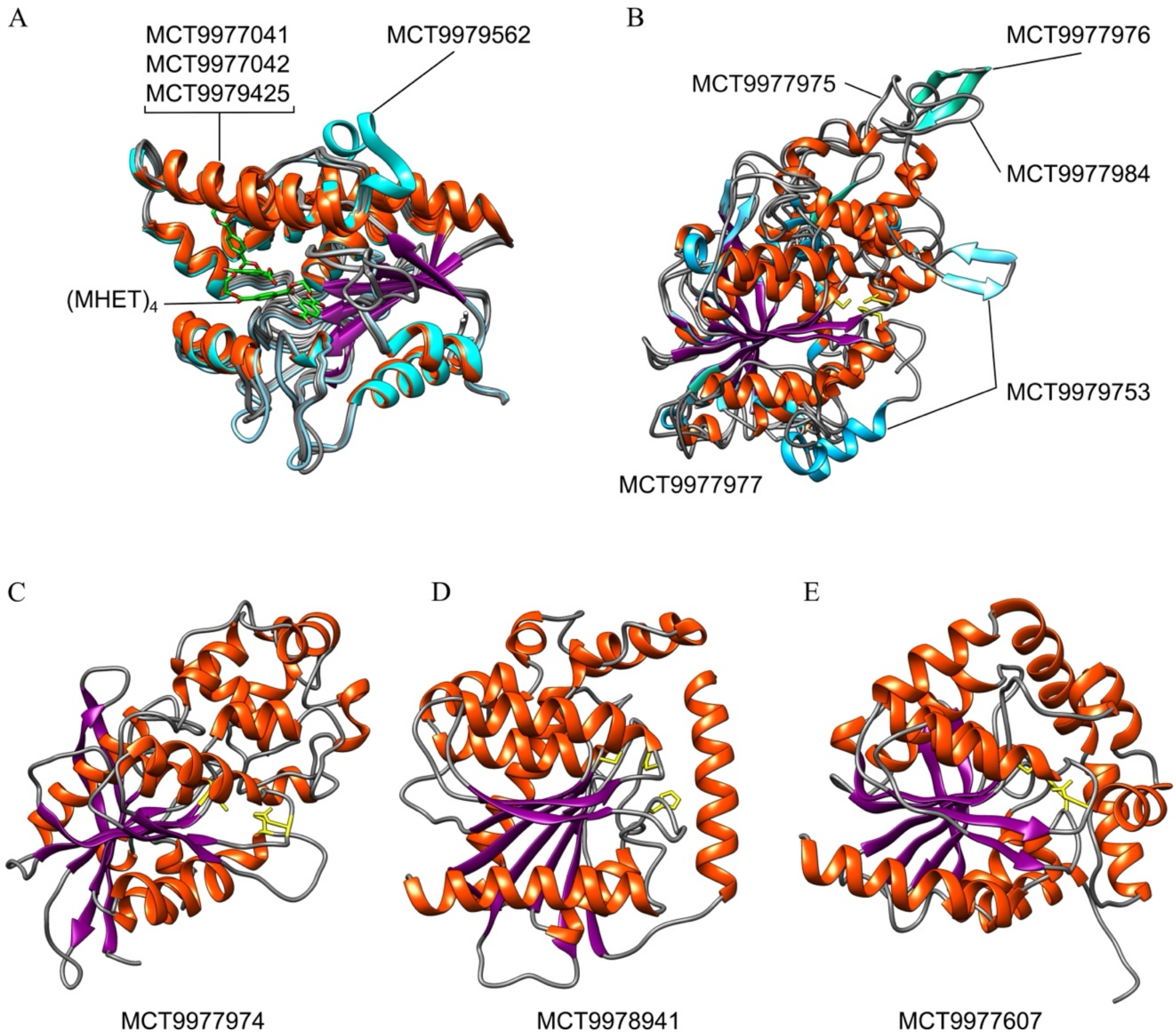

Figure S5. SDS–PAGE analysis of recombinant lipase expression in *E. coli*. Lipases MCT9979425, MCT9977041, MCT9977984, and MCT9977977 were analyzed after induction. For each construct, different purification fractions (T) and the resuspended cell pellet fraction (P) were loaded onto the gel. The majority of the recombinant proteins were detected in the pellet fractions, indicating that most of the expressed protein was present in the insoluble fraction. For lipase MCT9977977 expressed in *E. coli* C43, no distinct band corresponding to the expected molecular mass (~41 kDa) was observed, suggesting unsuccessful expression and/or induction under the tested conditions. Molecular weight markers (kDa) are indicated on both sides of the gel.

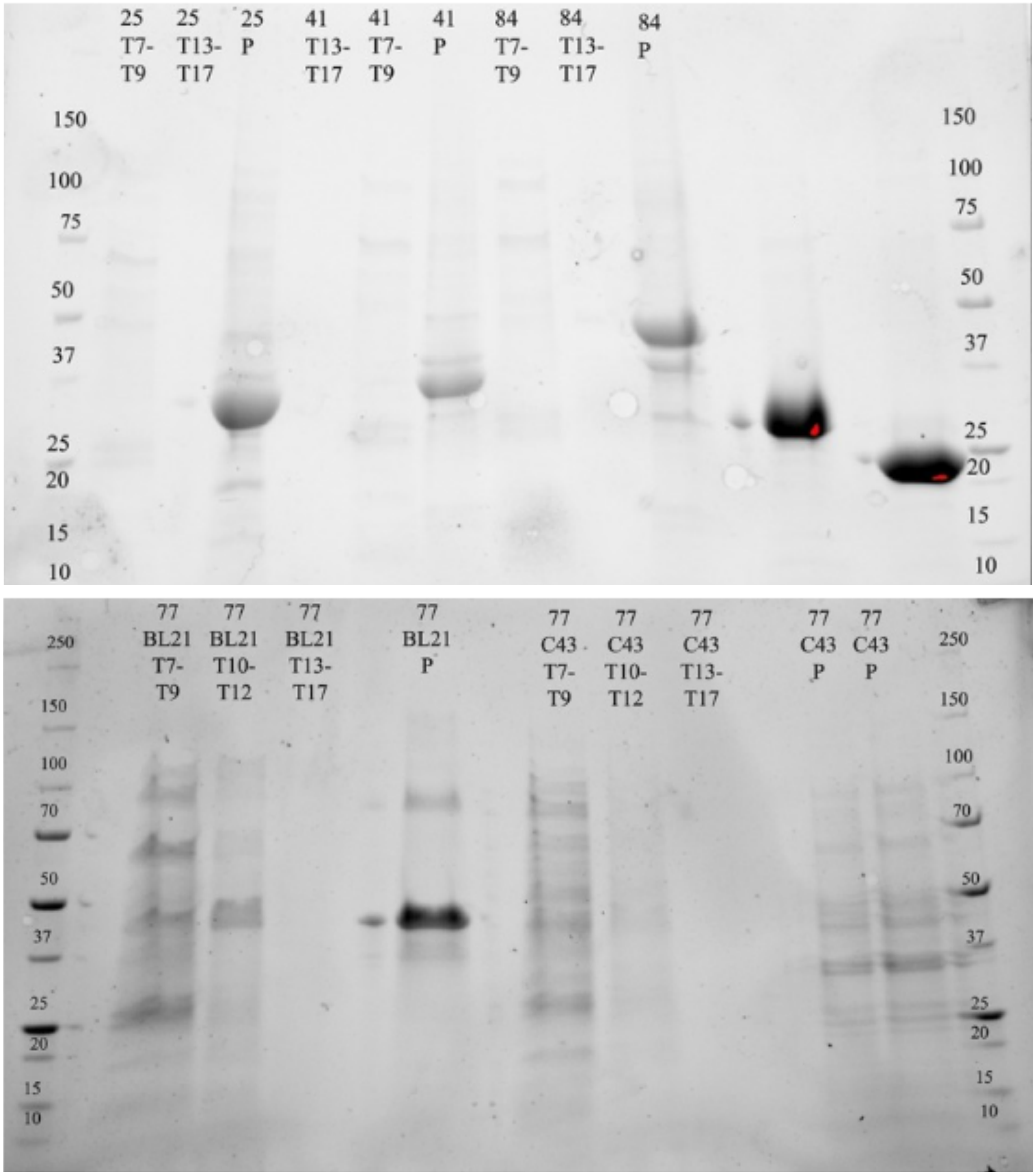

Figure S6. AlphaFold3 molecular models of putative lipases (MCT9979425, MCT9977041, MCT9977977 and MCT9977984), excluding signal peptides, and of a lipid transporter (MCT9980412).

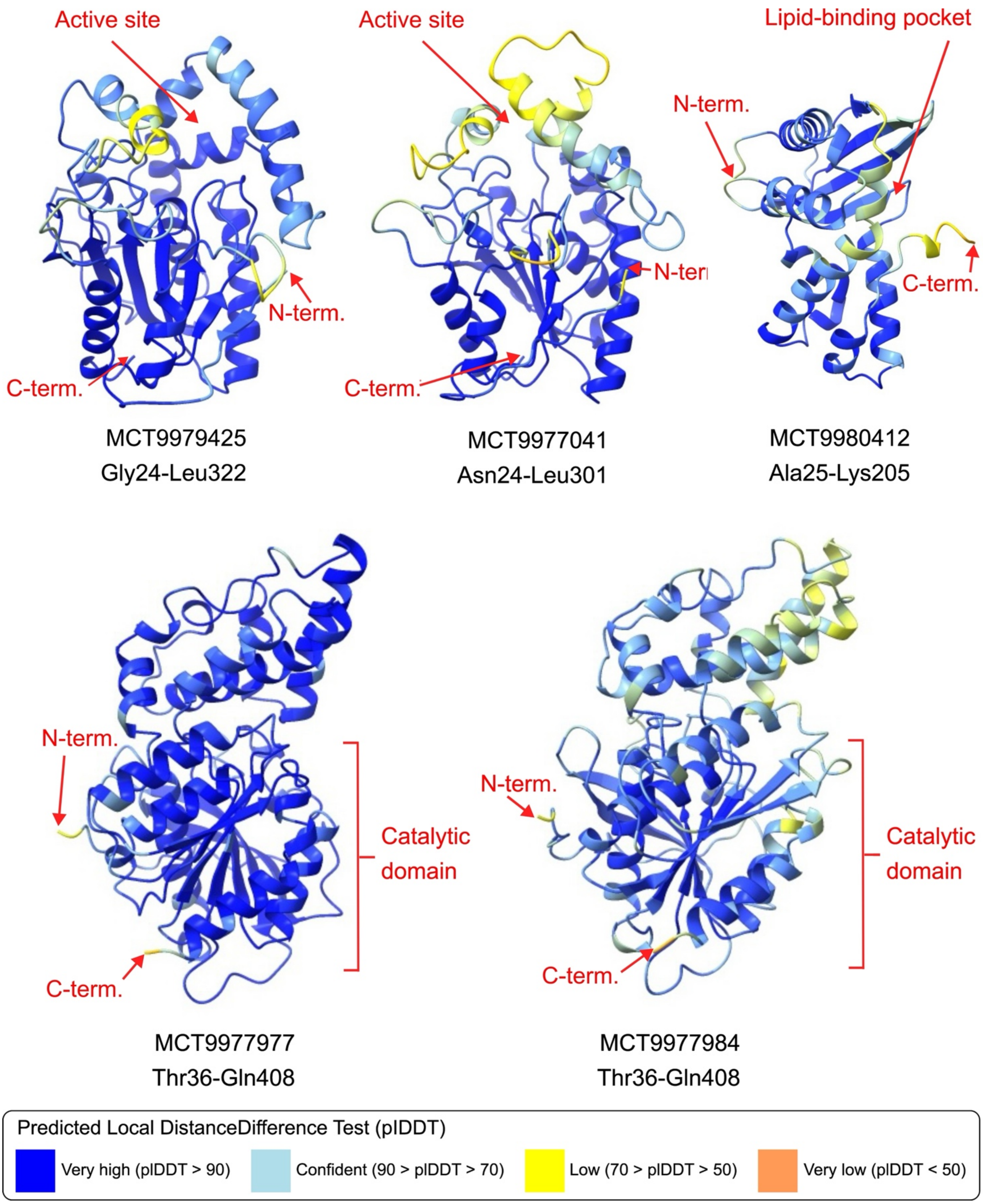

Table S1. Proteomic analysis.

Supernatant of *A. guillouiae* cultivation in MSM with 20 mg PET

Accession	Score	Mass	Num. of matches	Num. of significant matches	Num. of sequences	Num. of significant sequences	emPAI	Description
MCT9978521.1	317	13345	9	9	3	3	1.49	hypothetical M2R48_09305
MCT9979159.1	301	79677	12	12	4	4	0.23	malate synthase G
MCT9980077.1	252	12320	7	5	4	4	2.72	hypothetical M2R48_17265
MCT9976935.1	191	12664	11	9	6	6	5.82	50S ribosomal L7/L12
MCT9976744.1	190	36171	6	6	5	5	0.77	DUF6091 family
MCT9980412.1	179	22516	8	7	7	6	1.98	ABC transporter substrate-binding
MCT9977418.1	174	41150	2	2	1	1	0.11	NADH:flavin oxidoreductase/NADH oxidase
MCT9978037.1	160	12837	7	6	1	1	0.37	roadblock/LC7 domain-containing
MCT9979425.1	146	34961	5	5	1	1	0.13	triacylglycerol lipase
MCT9977078.1	122	24539	2	2	1	1	0.18	hypothetical M2R48_01850
MCT9979516.1	116	82222	6	5	2	2	0.11	NADP-dependent isocitrate dehydrogenase
MCT9980009.1	111	32563	4	4	4	4	0.66	amino acid ABC transporter substrate-binding
MCT9977977.1	109	42869	5	4	1	1	0.1	lipase family
MCT9978536.1	99	94490	4	3	2	2	0.09	bifunctional aconitate hydratase 2/2-methylisocitrate dehydratase
MCT9977041.1	97	34554	2	2	1	1	0.13	triacylglycerol lipase
MCT9980045.1	81	74127	3	3	1	1	0.06	methyl-accepting chemotaxis
MCT9977190.1	75	35205	2	2	2	2	0.26	malate dehydrogenase
MCT9976898.1	72	15349	3	3	2	2	0.7	DUF2147 domain-containing
MCT9979653.1	67	60160	2	2	1	1	0.07	NAD+ synthase
MCT9977579.1	64	44680	2	2	2	2	0.2	DcaP family trimeric outer membrane transporter
MCT9980041.1	62	4829	1	1	1	1	1.22	entericidin A/B family lipo
MCT9976929.1	62	48095	5	3	5	3	0.29	elongation factor Tu
MCT9979517.1	60	90950	1	1	1	1	0.05	acyl-CoA dehydrogenase
MCT9979254.1	58	7877	1	1	1	1	0.66	cold-shock
MCT9977830.1	58	18869	2	2	2	2	0.54	hypothetical M2R48_05775
MCT9977549.1	56	22541	1	1	1	1	0.2	OmpA family
MCT9977404.1	55	37255	5	2	4	2	0.25	OmpA family
MCT9980415.1	55	29723	1	1	1	1	0.15	ABC transporter ATP-binding
MCT9979583.1	54	37233	2	2	1	1	0.12	sulfate ABC transporter substrate-binding
MCT9980375.1	52	15380	1	1	1	1	0.3	50S ribosomal L15
MCT9979594.1	51	14402	3	2	3	2	0.76	hypothetical M2R48_14755
MCT9977738.1	51	13584	2	2	2	2	0.82	SCP2 sterol-binding domain-containing
MCT9978342.1	50	51378	1	1	1	1	0.08	adenylosuccinate lyase
MCT9980325.1	48	13476	1	1	1	1	0.35	cytochrome b562
MCT9980649.1	48	55426	1	1	1	1	0.08	F0F1 ATP synthase subunit alpha
MCT9976933.1	47	23769	1	1	1	1	0.19	50S ribosomal L1
MCT9976943.1	46	21955	1	1	1	1	0.2	outer membrane beta-barrel
MCT9977016.1	45	16329	3	3	3	3	1.12	pilin
MCT9977542.1	45	59239	4	2	3	2	0.15	isocitrate lyase
MCT9978035.1	43	11135	1	1	1	1	0.44	hypothetical M2R48_06820
MCT9980601.1	43	61647	1	1	1	1	0.07	long-chain-fatty-acid--CoA ligase
MCT9980647.1	41	50142	2	2	1	1	0.09	F0F1 ATP synthase subunit beta
MCT9979227.1	41	20464	1	1	1	1	0.22	YceI family
MCT9979609.1	40	26704	1	1	1	1	0.17	electron transfer flavo subunit beta/FixA family
MCT9980078.1	39	102315	1	1	1	1	0.04	pre translocase subunit SecA
MCT9977492.1	39	26953	1	1	1	1	0.16	response regulator transcription factor BfmR
MCT9980715.1	37	42110	1	1	1	1	0.1	NAD-dependent epimerase/dehydratase family
MCT9980017.1	35	61328	1	1	1	1	0.07	oleate hydratase
MCT9976754.1	31	20452	2	2	2	2	0.49	peptidylprolyl isomerase
MCT9976954.1	30	77966	1	1	1	1	0.05	fatty acid oxidation complex subunit alpha FadB
MCT9977609.1	29	53256	1	1	1	1	0.08	long-chain fatty acid transporter
MCT9976733.1	29	13286	1	1	1	1	0.36	NirD/YgiW/YdeI family stress tolerance

MCT9980748.1	29	11783	1	1	1	1	0.41	RcnB family
MCT9980369.1	28	37152	1	1	1	1	0.12	DNA-directed RNA polymerase subunit alpha
MCT9978676.1	28	9244	3	1	3	1	0.55	HU family DNA-binding
MCT9976862.1	25	5853	1	1	1	1	0.95	hypothetical M2R48_00740
MCT9979661.1	25	17504	1	1	1	1	0.26	hypothetical M2R48_15100
MCT9980382.1	18	19965	1	1	1	1	0.23	50S ribosomal L5
MCT9978717.1	18	43126	1	1	1	1	0.1	phosphotransferase family
MCT9980081.1	15	8677	1	1	1	1	0.59	acyl carrier

Supernatant of *A. guillouiae* cultivation in MSM with 20 mg PET supplemented with 100 mg tween 80.

Accession	Score	Mass	Num. of matches	Num. of significant matches	Num. of sequences	Num. of significant sequences	emPAI	Description
MCT9980767.1	740	18759	39	27	11	10	12.65	type VI secretion system tube Hcp
MCT9979159.1	425	79677	13	11	5	5	0.3	malate synthase G
MCT9979883.1	360	59763	12	10	8	6	0.41	bifunctional aspartate transaminase/aspartate 4-decarboxylase
MCT9976935.1	348	12664	14	14	7	7	8.4	50S ribosomal L7/L12
MCT9979425.1	345	34961	12	10	6	6	1.28	triacylglycerol lipase
MCT9979372.1	344	115362	13	11	8	7	0.29	type I secretion C-terminal target domain-containing , partial
MCT9976929.1	337	48095	16	13	8	7	0.83	elongation factor Tu
MCT9977951.1	301	24288	4	4	2	2	0.4	hypothetical M2R48_06385
MCT9979254.1	291	7877	6	6	2	2	1.75	cold-shock
MCT9978037.1	285	12837	9	8	2	2	0.88	roadblock/LC7 domain-containing
MCT9977041.1	281	34554	8	8	5	5	0.82	triacylglycerol lipase
MCT9977016.1	262	16329	7	7	3	3	1.12	pilin
MCT9977190.1	222	35205	5	5	4	4	0.8	malate dehydrogenase
MCT9977977.1	210	42869	6	6	2	2	0.21	lipase family
MCT9979635.1	198	41621	6	5	4	3	0.35	ADP-forming succinate--CoA ligase subunit beta
MCT9977390.1	192	11187	5	5	3	3	1.95	integration host factor subunit alpha
MCT9976934.1	191	18191	3	3	1	1	0.25	50S ribosomal L10
MCT9977492.1	191	26953	7	5	3	2	0.36	response regulator transcription factor BfmR
MCT9978536.1	179	94490	8	6	3	2	0.09	bifunctional aconitate hydratase 2/2-methylisocitrate dehydratase
MCT9980045.1	160	74127	3	2	1	1	0.06	methyl-accepting chemotaxis
MCT9979638.1	150	105478	5	4	3	2	0.08	2-oxoglutarate dehydrogenase E1 component
MCT9978676.1	146	9244	8	6	5	4	4.7	HU family DNA-binding
MCT9979653.1	138	60160	2	2	2	2	0.15	NAD+ synthase
MCT9979634.1	128	30874	4	3	2	2	0.31	succinate--CoA ligase subunit alpha
MCT9979301.1	121	15716	2	2	2	2	0.68	50S ribosomal L9
MCT9979954.1	121	79043	6	4	5	3	0.17	elongation factor G
MCT9977984.1	117	45102	3	2	1	1	0.1	lipase family
MCT9979516.1	105	82222	4	3	1	1	0.05	NADP-dependent isocitrate dehydrogenase
MCT9977542.1	100	59239	6	4	3	2	0.15	isocitrate lyase
MCT9979562.1	97	36366	4	4	3	3	0.4	triacylglycerol lipase
MCT9978565.1	97	33264	2	2	1	1	0.13	ParB/RepB/Spo0J family partition
MCT9980601.1	95	61647	2	2	2	2	0.14	long-chain-fatty-acid--CoA ligase
MCT9976923.1	93	48026	1	1	1	1	0.09	phosphoglucosamine mutase
MCT9980009.1	91	32563	5	4	4	4	0.66	amino acid ABC transporter substrate-binding
MCT9980392.1	90	21457	3	3	2	2	0.47	50S ribosomal L4
MCT9980235.1	87	36746	2	2	2	2	0.25	ketol-acid reductoisomerase
MCT9976933.1	86	23769	3	2	2	2	0.41	50S ribosomal L1
MCT9980041.1	85	4829	3	2	1	1	1.22	entericidin A/B family lipo
MCT9980771.1	84	99271	1	1	1	1	0.04	type VI secretion system ATPase TssH
MCT9976953.1	84	41400	4	3	2	2	0.22	acetyl-CoA C-acyltransferase FadA
MCT9980715.1	81	42110	2	2	2	2	0.22	NAD-dependent epimerase/dehydratase family
MCT9977420.1	80	28954	1	1	1	1	0.15	sel1 repeat family
MCT9980394.1	78	11709	2	2	1	1	0.41	30S ribosomal S10
MCT9980229.1	77	15438	3	2	1	1	0.3	nucleoside-diphosphate kinase
MCT9977365.1	76	65167	3	2	3	2	0.14	acyl-CoA dehydrogenase C-terminal domain-containing
MCT9979621.1	76	48774	1	1	1	1	0.09	NADP-specific glutamate dehydrogenase
MCT9978781.1	76	20753	2	1	1	1	0.22	TerD family
MCT9980081.1	75	8677	2	1	1	1	0.59	acyl carrier
MCT9980064.1	74	6939	2	2	2	2	2.11	hypothetical M2R48_17180
MCT9977198.1	74	69803	2	1	2	1	0.06	hypothetical M2R48_02480
MCT9980738.1	73	26386	2	2	1	1	0.17	ribonuclease PH
MCT9977078.1	73	24539	2	2	1	1	0.18	hypothetical M2R48_01850
MCT9980770.1	72	23746	2	2	1	1	0.19	hypothetical M2R48_20840
MCT9977502.1	72	18901	1	1	1	1	0.24	NADH-quinone oxidoreductase subunit NuoE
MCT9979957.1	72	53816	2	2	1	1	0.08	EcsC family
MCT9978516.1	70	43684	1	1	1	1	0.1	formate-dependent phosphoribosylglycinamide formyltransferase
MCT9976847.1	70	17570	2	1	1	1	0.26	SCP2 sterol-binding domain-containing
MCT9979637.1	69	43471	5	3	4	3	0.33	2-oxoglutarate dehydrogenase complex dihydrolipoyllysine-residue succinyltransferase
MCT9980110.1	68	12434	3	3	3	3	1.66	YegP family

MCT9980397.1	68	56423	1	1	1	1	0.08	DUF853 domain-containing
MCT9980005.1	67	44652	2	1	2	1	0.1	UDP-N-acetylglucosamine 1-carboxyvinyltransferase
MCT9977939.1	67	30577	3	2	2	1	0.14	translation elongation factor Ts
MCT9977549.1	66	22541	4	4	2	2	0.44	OmpA family
MCT9978893.1	65	18589	2	2	1	1	0.25	peptidylprolyl isomerase
MCT9978616.1	65	84042	1	1	1	1	0.05	ATP-dependent Clp protease ATP-binding subunit ClpA
MCT9977214.1	64	22572	1	1	1	1	0.2	RdgB/HAM1 family non-canonical purine NTP pyrophosphatase
MCT9980472.1	64	74736	2	2	2	2	0.12	polyribonucleotide nucleotidyltransferase
MCT9978748.1	64	43081	2	1	1	1	0.1	FAD-dependent oxidoreductase
MCT9977418.1	63	41150	1	1	1	1	0.11	NADH:flavin oxidoreductase/NADH oxidase
MCT9977738.1	62	13584	2	2	1	1	0.35	SCP2 sterol-binding domain-containing
MCT9980518.1	62	38993	2	2	1	1	0.11	pilus assembly PilM
MCT9979512.1	60	45576	2	1	2	1	0.09	NADP-dependent isocitrate dehydrogenase
MCT9980370.1	60	23210	3	3	3	3	0.7	30S ribosomal S4
MCT9979608.1	60	31385	6	2	6	2	0.3	FAD-binding
MCT9978322.1	59	25171	1	1	1	1	0.18	alpha/beta hydrolase
MCT9978779.1	58	20438	2	2	1	1	0.22	TerD family
MCT9979230.1	58	71488	1	1	1	1	0.06	transketolase
MCT9978049.1	57	83336	1	1	1	1	0.05	Ig-like repeat Blp2
MCT9980649.1	55	55426	5	3	4	3	0.25	F0F1 ATP synthase subunit alpha
MCT9978342.1	55	51378	1	1	1	1	0.08	adenylosuccinate lyase
MCT9980390.1	54	30203	1	1	1	1	0.15	50S ribosomal L2
MCT9977704.1	53	57147	2	1	2	1	0.08	chaperonin GroEL
MCT9979023.1	52	52434	1	1	1	1	0.08	type I glutamate--ammonia ligase
MCT9978672.1	52	44913	1	1	1	1	0.1	IscS subfamily cysteine desulfurase
MCT9977404.1	50	37255	1	1	1	1	0.12	OmpA family
MCT9977766.1	49	102468	1	1	1	1	0.04	restriction endonuclease
MCT9980383.1	47	11149	1	1	1	1	0.44	50S ribosomal L24
MCT9977934.1	46	45245	1	1	1	1	0.1	NADH:flavin oxidoreductase/NADH oxidase family
MCT9980639.1	46	18316	4	2	3	2	0.56	hypothetical M2R48_20165
MCT9977241.1	45	80383	1	1	1	1	0.05	carboxy terminal-processing peptidase
MCT9978888.1	42	16940	1	1	1	1	0.27	SRPBCC domain-containing
MCT9978450.1	42	54003	1	1	1	1	0.08	OmpA family
MCT9980573.1	41	45986	2	2	2	2	0.2	aminotransferase class I/II-fold pyridoxal phosphate-dependent enzyme
MCT9978035.1	41	11135	1	1	1	1	0.44	hypothetical M2R48_06820
MCT9980375.1	40	15380	2	2	2	2	0.69	50S ribosomal L15
MCT9980080.1	40	16777	3	3	2	2	0.63	peptidoglycan-binding LysM
MCT9979609.1	40	26704	4	1	4	1	0.17	electron transfer flavo subunit beta/FixA family
MCT9976862.1	40	5853	1	1	1	1	0.95	hypothetical M2R48_00740
MCT9978825.1	39	15866	1	1	1	1	0.29	universal stress
MCT9977220.1	39	62687	1	1	1	1	0.07	2-isopropylmalate synthase
MCT9976827.1	38	26862	1	1	1	1	0.17	phosphoribosylaminoimidazolesuccinocarboxamide synthase
MCT9977161.1	37	10275	1	1	1	1	0.48	hypothetical M2R48_02295
MCT9980173.1	37	14732	1	1	1	1	0.32	organic hydroperoxide resistance
MCT9980510.1	37	162618	4	3	2	2	0.05	glutamate synthase large subunit
MCT9979180.1	36	10130	1	1	1	1	0.49	YARHG domain-containing
MCT9978442.1	36	94765	2	1	2	1	0.04	ATP-dependent chaperone ClpB
MCT9980647.1	36	50142	3	1	2	1	0.09	F0F1 ATP synthase subunit beta
MCT9978283.1	35	192920	1	1	1	1	0.02	RHS domain-containing
MCT9978004.1	35	40440	1	1	1	1	0.11	amidohydrolase family
MCT9979289.1	34	86566	2	1	2	1	0.05	phosphoenolpyruvate synthase
MCT9980412.1	33	22516	1	1	1	1	0.2	ABC transporter substrate-binding
MCT9976876.1	33	59469	1	1	1	1	0.07	glutamate--cysteine ligase
MCT9978644.1	33	41139	2	1	1	1	0.11	phosphoglycerate kinase
MCT9978508.1	32	17956	1	1	1	1	0.25	glutathione peroxidase
MCT9979316.1	30	56021	1	1	1	1	0.08	bifunctional phosphoribosylaminoimidazolecarboxamide formyltransferase/IMP cyclohydrolase
MCT9978585.1	30	61292	6	2	6	2	0.14	30S ribosomal S1
MCT9980084.1	30	7106	1	1	1	1	0.75	50S ribosomal L32
MCT9977107.1	29	57287	1	1	1	1	0.07	catalase
MCT9978080.1	27	87120	2	1	2	1	0.05	CSLREA domain-containing
MCT9980522.1	25	50742	1	1	1	1	0.08	UDP-N-acetylmuramoyl-tripeptide--D-alanyl-D-alanine ligase
MCT9978824.1	25	16633	1	1	1	1	0.28	universal stress
MCT9977052.1	23	43886	1	1	1	1	0.1	phosphoglycerate dehydrogenase

MCT9980202.1	23	32581	1	1	1	1	0.13	RNase adapter RapZ
MCT9979379.1	21	35584	1	1	1	1	0.12	KpsF/GutQ family sugar-phosphate isomerase
MCT9979955.1	21	17674	1	1	1	1	0.26	30S ribosomal S7
MCT9977529.1	21	34908	1	1	1	1	0.13	sulfate adenylyltransferase subunit CysD
MCT9979690.1	19	33620	1	1	1	1	0.13	LysR substrate-binding domain-containing
MCT9980398.1	18	44077	1	1	1	1	0.1	NO-inducible flavohemo
MCT9980761.1	17	36694	1	1	1	1	0.12	type VI secretion system-associated TagF
MCT9977393.1	17	11691	1	1	1	1	0.41	thioredoxin
MCT9980391.1	16	11567	1	1	1	1	0.42	50S ribosomal L23
MCT9977449.1	14	16275	1	1	1	1	0.28	DUF2147 domain-containing
MCT9980768.1	13	55288	1	1	1	1	0.08	type VI secretion system contractile sheath large subunit
MCT9980622.1	13	28951	3	1	2	1	0.15	hypothetical M2R48_20075

Supernatant of *A. guillouiae* cultivation in MSM with 20 mg PET supplemented with 200 mg tween 80.

Accession	Score	Mass	Num. of matches	Num. of significant matches	Num. of sequences	Num. of significant sequences	emPAI	Description
MCT9979425.1	616	34961	14	12	8	7	1.28	triacylglycerol lipase
MCT9977977.1	328	42869	8	7	4	3	0.34	lipase family
MCT9978037.1	302	12837	11	10	2	2	0.88	roadblock/LC7 domain-containing
MCT9977016.1	267	16329	9	9	5	5	3.47	pilin
MCT9977984.1	261	45102	10	8	4	3	0.32	lipase family
MCT9977041.1	246	34554	8	7	6	5	0.82	triacylglycerol lipase
MCT9976935.1	245	12664	11	9	6	5	3.96	50S ribosomal L7/L12
MCT9979159.1	207	79677	8	8	3	3	0.17	malate synthase G
MCT9976929.1	203	48095	9	8	5	4	0.41	elongation factor Tu
MCT9980649.1	159	55426	4	3	3	2	0.16	F0F1 ATP synthase subunit alpha
MCT9977404.1	159	37255	5	5	4	4	0.56	OmpA family
MCT9979635.1	140	41621	4	3	3	3	0.35	ADP-forming succinate--CoA ligase subunit beta
MCT9978521.1	140	13345	6	6	3	3	1.49	hypothetical M2R48_09305
MCT9977542.1	139	59239	3	3	1	1	0.07	isocitrate lyase
MCT9979638.1	119	105478	4	3	3	2	0.08	2-oxoglutarate dehydrogenase E1 component
MCT9976953.1	118	41400	5	3	4	3	0.35	acetyl-CoA C-acyltransferase FadA
MCT9977492.1	106	26953	4	2	3	1	0.16	response regulator transcription factor BfmR
MCT9980601.1	106	61647	5	2	2	1	0.07	long-chain-fatty-acid--CoA ligase
MCT9978035.1	103	11135	2	2	2	2	1.07	hypothetical M2R48_06820
MCT9980009.1	103	32563	4	3	4	3	0.46	amino acid ABC transporter substrate-binding
MCT9976934.1	99	18191	2	2	1	1	0.25	50S ribosomal L10
MCT9979637.1	96	43471	6	3	3	1	0.1	2-oxoglutarate dehydrogenase complex dihydrolipoyllysine-residue succinyltransferase
MCT9979254.1	92	7877	2	2	1	1	0.66	cold-shock
MCT9979609.1	86	26704	1	1	1	1	0.17	electron transfer flavo subunit beta/FixA family
MCT9980045.1	85	74127	1	1	1	1	0.06	methyl-accepting chemotaxis
MCT9979023.1	85	52434	2	2	1	1	0.08	type I glutamate--ammonia ligase
MCT9979621.1	76	48774	1	1	1	1	0.09	NADP-specific glutamate dehydrogenase
MCT9980229.1	74	15438	4	3	2	2	0.69	nucleoside-diphosphate kinase
MCT9978049.1	72	83336	1	1	1	1	0.05	Ig-like repeat Blp2
MCT9978676.1	70	9244	3	3	2	2	1.39	HU family DNA-binding
MCT9980767.1	69	18759	2	2	1	1	0.24	type VI secretion system tube Hcp
MCT9976933.1	68	23769	4	4	3	3	0.68	50S ribosomal L1
MCT9979954.1	67	79043	2	2	2	2	0.11	elongation factor G
MCT9977951.1	67	24288	1	1	1	1	0.18	hypothetical M2R48_06385
MCT9980064.1	66	6939	3	2	3	2	2.11	hypothetical M2R48_17180
MCT9979634.1	64	30874	6	6	3	3	0.49	succinate--CoA ligase subunit alpha
MCT9978501.1	63	8446	2	1	2	1	0.61	30S ribosomal S21
MCT9980375.1	62	15380	3	3	3	3	1.21	50S ribosomal L15
MCT9978710.1	60	53877	1	1	1	1	0.08	aminotransferase class III-fold pyridoxal phosphate-dependent enzyme
MCT9977579.1	60	44680	1	1	1	1	0.1	DcaP family trimeric outer membrane transporter
MCT9979530.1	60	82277	1	1	1	1	0.05	NADP-dependent malic enzyme
MCT9980394.1	56	11709	1	1	1	1	0.41	30S ribosomal S10
MCT9978536.1	55	94490	2	1	2	1	0.04	bifunctional aconitate hydratase 2/2-methylisocitrate dehydratase
MCT9979372.1	54	115362	2	1	2	1	0.04	type I secretion C-terminal target domain-containing , partial
MCT9977241.1	53	80383	1	1	1	1	0.05	carboxy terminal-processing peptidase
MCT9980110.1	53	12434	2	2	1	1	0.38	YegP family
MCT9980390.1	52	30203	1	1	1	1	0.15	50S ribosomal L2
MCT9976898.1	52	15349	3	2	3	2	0.7	DUF2147 domain-containing
MCT9978080.1	51	87120	2	1	2	1	0.05	CSLREA domain-containing
MCT9980081.1	50	8677	1	1	1	1	0.59	acyl carrier
MCT9980325.1	50	13476	1	1	1	1	0.35	cytochrome b562
MCT9978043.1	49	20681	2	2	2	2	0.49	alkyl hydroperoxide reductase subunit C
MCT9977390.1	49	11187	1	1	1	1	0.43	integration host factor subunit alpha
MCT9977365.1	48	65167	2	1	2	1	0.07	acyl-CoA dehydrogenase C-terminal domain-containing
MCT9977549.1	48	22541	2	2	2	2	0.44	OmpA family
MCT9980389.1	47	10189	2	2	1	1	0.48	30S ribosomal S19
MCT9979180.1	46	10130	2	2	1	1	0.49	YARHG domain-containing
MCT9980715.1	45	42110	1	1	1	1	0.1	NAD-dependent epimerase/dehydratase family
MCT9979517.1	45	90950	1	1	1	1	0.05	acyl-CoA dehydrogenase

MCT9979594.1	44	14402	1	1	1	1	0.33	hypothetical M2R48_14755
MCT9980041.1	44	4829	2	1	2	1	1.22	entericidin A/B family lipo
MCT9977078.1	42	24539	1	1	1	1	0.18	hypothetical M2R48_01850
MCT9980738.1	42	26386	2	1	1	1	0.17	ribonuclease PH
MCT9979316.1	42	56021	1	1	1	1	0.08	bifunctional phosphoribosylaminoimidazolecarboxamide formyltransferase/IMP cyclohydrolase
MCT9980378.1	41	12456	1	1	1	1	0.38	50S ribosomal L18
MCT9979379.1	41	35584	1	1	1	1	0.12	KpsF/GutQ family sugar-phosphate isomerase
MCT9979562.1	39	36366	1	1	1	1	0.12	triacylglycerol lipase
MCT9980080.1	39	16777	2	1	2	1	0.28	peptidoglycan-binding LysM
MCT9977107.1	38	57287	1	1	1	1	0.07	catalase
MCT9980056.1	38	29534	2	1	2	1	0.15	DUF4882 family
MCT9978587.1	38	13531	1	1	1	1	0.35	lipopolysaccharide assembly LapA domain-containing
MCT9976862.1	37	5853	2	1	2	1	0.95	hypothetical M2R48_00740
MCT9980371.1	34	13526	2	2	2	2	0.82	30S ribosomal S11
MCT9980383.1	34	11149	1	1	1	1	0.44	50S ribosomal L24
MCT9976744.1	33	36171	1	1	1	1	0.12	DUF6091 family
MCT9980084.1	32	7106	1	1	1	1	0.75	50S ribosomal L32
MCT9980734.1	32	42953	1	1	1	1	0.1	hypothetical M2R48_20660
MCT9977443.1	30	15960	1	1	1	1	0.29	50S ribosomal L13
MCT9979640.1	30	69333	1	1	1	1	0.06	succinate dehydrogenase flavo subunit
MCT9979608.1	30	31385	6	3	3	2	0.3	FAD-binding
MCT9980639.1	29	18316	2	1	2	1	0.25	hypothetical M2R48_20165
MCT9976954.1	29	77966	2	2	1	1	0.05	fatty acid oxidation complex subunit alpha FadB
MCT9977225.1	28	49593	1	1	1	1	0.09	trigger factor
MCT9980078.1	28	102315	1	1	1	1	0.04	pre translocase subunit SecA
MCT9978283.1	28	192920	3	2	1	1	0.02	RHS domain-containing
MCT9980262.1	27	35335	1	1	1	1	0.12	pirin family
MCT9980372.1	25	13202	1	1	1	1	0.36	30S ribosomal S13
MCT9978644.1	25	41139	1	1	1	1	0.11	phosphoglycerate kinase
MCT9979301.1	24	15716	2	2	2	2	0.68	50S ribosomal L9
MCT9979956.1	23	13801	1	1	1	1	0.34	30S ribosomal S12
MCT9980412.1	22	22516	1	1	1	1	0.2	ABC transporter substrate-binding
MCT9979688.1	20	42161	1	1	1	1	0.1	FAD-dependent oxidoreductase
MCT9977034.1	20	127159	1	1	1	1	0.03	PilC/PilY family type IV pilus
MCT9980370.1	19	23210	2	1	2	1	0.19	30S ribosomal S4
MCT9980549.1	18	28712	1	1	1	1	0.15	two-component system response regulator OmpR
MCT9980173.1	18	14732	1	1	1	1	0.32	organic hydroperoxide resistance
MCT9980379.1	17	19159	2	1	1	1	0.24	50S ribosomal L6
MCT9980622.1	17	28951	1	1	1	1	0.15	hypothetical M2R48_20075
MCT9977248.1	13	100907	1	1	1	1	0.04	aconitate hydratase AcnA

Supernatant of *A. guillouiae* cultivation in MSM with 200 mg tween 80.

Accession	Score	Mass	Num. of matches	Num. of significant matches	Num. of sequences	Num. of significant sequences	emPAI	Description
MCT9978521.1	317	13345	9	9	3	3	1.49	hypothetical M2R48_09305
MCT9979159.1	301	79677	12	12	4	4	0.23	malate synthase G
MCT9980077.1	252	12320	7	5	4	4	2.72	hypothetical M2R48_17265
MCT9976935.1	191	12664	11	9	6	6	5.82	50S ribosomal L7/L12
MCT9976744.1	190	36171	6	6	5	5	0.77	DUF6091 family
MCT9980412.1	179	22516	8	7	7	6	1.98	ABC transporter substrate-binding
MCT9977418.1	174	41150	2	2	1	1	0.11	NADH:flavin oxidoreductase/NADH oxidase
MCT9978037.1	160	12837	7	6	1	1	0.37	roadblock/LC7 domain-containing
MCT9979425.1	146	34961	5	5	1	1	0.13	triacylglycerol lipase
MCT9977078.1	122	24539	2	2	1	1	0.18	hypothetical M2R48_01850
MCT9979516.1	116	82222	6	5	2	2	0.11	NADP-dependent isocitrate dehydrogenase
MCT9980009.1	111	32563	4	4	4	4	0.66	amino acid ABC transporter substrate-binding
MCT9977977.1	109	42869	5	4	1	1	0.1	lipase family
MCT9978536.1	99	94490	4	3	2	2	0.09	bifunctional aconitate hydratase 2/2-methylisocitrate dehydratase
MCT9977041.1	97	34554	2	2	1	1	0.13	triacylglycerol lipase
MCT9980045.1	81	74127	3	3	1	1	0.06	methyl-accepting chemotaxis
MCT9977190.1	75	35205	2	2	2	2	0.26	malate dehydrogenase
MCT9976898.1	72	15349	3	3	2	2	0.7	DUF2147 domain-containing
MCT9979653.1	67	60160	2	2	1	1	0.07	NAD+ synthase
MCT9977579.1	64	44680	2	2	2	2	0.2	DcaP family trimeric outer membrane transporter
MCT9980041.1	62	4829	1	1	1	1	1.22	entericidin A/B family lipo
MCT9976929.1	62	48095	5	3	5	3	0.29	elongation factor Tu
MCT9979517.1	60	90950	1	1	1	1	0.05	acyl-CoA dehydrogenase
MCT9979254.1	58	7877	1	1	1	1	0.66	cold-shock
MCT9977830.1	58	18869	2	2	2	2	0.54	hypothetical M2R48_05775
MCT9977549.1	56	22541	1	1	1	1	0.2	OmpA family
MCT9977404.1	55	37255	5	2	4	2	0.25	OmpA family
MCT9980415.1	55	29723	1	1	1	1	0.15	ABC transporter ATP-binding
MCT9979583.1	54	37233	2	2	1	1	0.12	sulfate ABC transporter substrate-binding
MCT9980375.1	52	15380	1	1	1	1	0.3	50S ribosomal L15
MCT9979594.1	51	14402	3	2	3	2	0.76	hypothetical M2R48_14755
MCT9977738.1	51	13584	2	2	2	2	0.82	SCP2 sterol-binding domain-containing
MCT9978342.1	50	51378	1	1	1	1	0.08	adenylosuccinate lyase
MCT9980325.1	48	13476	1	1	1	1	0.35	cytochrome b562
MCT9980649.1	48	55426	1	1	1	1	0.08	F0F1 ATP synthase subunit alpha
MCT9976933.1	47	23769	1	1	1	1	0.19	50S ribosomal L1
MCT9976943.1	46	21955	1	1	1	1	0.2	outer membrane beta-barrel
MCT9977016.1	45	16329	3	3	3	3	1.12	pilin
MCT9977542.1	45	59239	4	2	3	2	0.15	isocitrate lyase
MCT9978035.1	43	11135	1	1	1	1	0.44	hypothetical M2R48_06820
MCT9980601.1	43	61647	1	1	1	1	0.07	long-chain-fatty-acid--CoA ligase
MCT9980647.1	41	50142	2	2	1	1	0.09	F0F1 ATP synthase subunit beta
MCT9979227.1	41	20464	1	1	1	1	0.22	YceI family
MCT9979609.1	40	26704	1	1	1	1	0.17	electron transfer flavo subunit beta/FixA family
MCT9980078.1	39	102315	1	1	1	1	0.04	pre translocase subunit SecA
MCT9977492.1	39	26953	1	1	1	1	0.16	response regulator transcription factor BfmR
MCT9980715.1	37	42110	1	1	1	1	0.1	NAD-dependent epimerase/dehydratase family
MCT9980017.1	35	61328	1	1	1	1	0.07	oleate hydratase
MCT9976754.1	31	20452	2	2	2	2	0.49	peptidylprolyl isomerase
MCT9976954.1	30	77966	1	1	1	1	0.05	fatty acid oxidation complex subunit alpha FadB
MCT9977609.1	29	53256	1	1	1	1	0.08	long-chain fatty acid transporter
MCT9976733.1	29	13286	1	1	1	1	0.36	NirD/YgiW/YdeI family stress tolerance
MCT9980748.1	29	11783	1	1	1	1	0.41	RcnB family
MCT9980369.1	28	37152	1	1	1	1	0.12	DNA-directed RNA polymerase subunit alpha

MCT9978676.1	28	9244	3	1	3	1	0.55	HU family DNA-binding
MCT9976862.1	25	5853	1	1	1	1	0.95	hypothetical M2R48_00740
MCT9979661.1	25	17504	1	1	1	1	0.26	hypothetical M2R48_15100
MCT9980382.1	18	19965	1	1	1	1	0.23	50S ribosomal L5
MCT9978717.1	18	43126	1	1	1	1	0.1	phosphotransferase family
MCT9980081.1	15	8677	1	1	1	1	0.59	acyl carrier
